# Differential Trafficking and Expression of PIR Proteins in Acute and Chronic *Plasmodium* Infections

**DOI:** 10.3389/fcimb.2022.877253

**Published:** 2022-06-16

**Authors:** Maria Giorgalli, Deirdre A. Cunningham, Malgorzata Broncel, Aaron Sait, Thomas E. Harrison, Caroline Hosking, Audrey Vandomme, Sarah I. Amis, Ana Antonello, Lauren Sullivan, Faith Uwadiae, Laura Torella, Matthew K. Higgins, Jean Langhorne

**Affiliations:** ^1^ Malaria Immunology Laboratory, The Francis Crick Institute, London, United Kingdom; ^2^ Proteomics Science Technology Platform, The Francis Crick Institute, London, United Kingdom; ^3^ Electron Microscopy Science Technology Platform, The Francis Crick Institute, London, United Kingdom; ^4^ Laboratory of Molecular Parasitology, Department of Biochemistry, University of Oxford, Oxford, United Kingdom

**Keywords:** malaria, *Plasmodium*, multigene families, *pir* genes, acute infection, chronic infection

## Abstract

*Plasmodium* multigene families are thought to play important roles in the pathogenesis of malaria. *Plasmodium interspersed repeat (pir)* genes comprise the largest multigene family in many *Plasmodium* species. However, their expression pattern and localisation remain to be elucidated. Understanding protein subcellular localisation is fundamental to reveal the functional importance and cell-cell interactions of the PIR proteins. Here, we use the rodent malaria parasite*, Plasmodium chabaudi chabaudi*, as a model to investigate the localisation pattern of this gene family. We found that most PIR proteins are co-expressed in clusters during acute and chronic infection; members of the S7 clade are predominantly expressed during the acute-phase, whereas members of the L1 clade dominate the chronic-phase of infection. Using peptide antisera specific for S7 or L1 PIRS, we show that these PIRs have different localisations within the infected red blood cells. S7 PIRs are exported into the infected red blood cell cytoplasm where they are co-localised with parasite-induced host cell modifications termed Maurer’s clefts, whereas L1 PIRs are localised on or close to the parasitophorous vacuolar membrane. This localisation pattern changes following mosquito transmission and during progression from acute- to chronic-phase of infection. The presence of PIRs in Maurer’s clefts, as seen for *Plasmodium falciparum* RIFIN and STEVOR proteins, might suggest trafficking of the PIRs on the surface of the infected erythrocytes. However, neither S7 nor L1 PIR proteins detected by the peptide antisera are localised on the surface of infected red blood cells, suggesting that they are unlikely to be targets of surface variant-specific antibodies or to be directly involved in adhesion of infected red blood cells to host cells, as described for *Plasmodium falciparum* VAR proteins. The differences in subcellular localisation of the two major clades of *Plasmodium chabaudi* PIRs across the blood cycle, and the apparent lack of expression on the red cell surface strongly suggest that the function(s) of this gene family may differ from those of other multigene families of *Plasmodium*, such as the *var* genes of *Plasmodium falciparum*.

## 1 Introduction

*Plasmodium* multigene families are thought to play important roles in malaria pathogenesis and immune evasion. The largest multigene family is the *Plasmodium interspersed repeat* (*pir*) gene family. This gene family is present in the genomes of many *Plasmodium* species including those infecting rodents and human, with the exception of the Laverania subgenus such as *Plasmodium falciparum* (Janssen et al., 2002; Yam et al., 2016). The number of *pir* genes varies considerably between the species, from 134 members in *Plasmodium berghei*, up to 1949 members in *Plasmodium ovale curtisi* (Aurrecoechea et al., 2009; Rutledge et al., 2017). Unlike the most highly studied multigene family, the *var* gene family of *Plasmodium falciparum*, the members of which are thought to be involved in antigenic variation, immune evasion and sequestration to host tissues (Buffet et al., 2011; Scherf et al., 1998; Scherf et al., 2008), the function of *pirs* remains unknown. In contrast to the *var* genes (Scherf et al., 1998), multiple *pir* genes are expressed simultaneously in the same blood-stage parasite (Cunningham et al., 2009; Howick et al., 2019; Otto et al., 2014; Reid et al., 2018; Sa et al., 2020) suggesting that antigenic variation of proteins on the surface of infected red blood cells (iRBC) may not be one of the roles of PIRs.

To elucidate the function of PIRs, it is necessary to be able to study them in a tractable experimental model. *Plasmodium chabaudi* is a well-studied, robust model to investigate mechanisms of immune evasion and antigenic variation, in particular with regards to the *pir* genes (Lamb et al., 2006; Langhorne et al., 2008). There are approximately 200 *pir* genes in *Plasmodium chabaudi chabaudi*. In the AS strain, the most commonly established laboratory strain, parasites isolated from the acute-phase of a mosquito-transmitted (MT) blood-stage infection have a different transcriptional profile of *pir* genes and show different virulence behaviour compared to those isolated at the chronic-phase of infection (Brugat et al., 2017; Spence et al., 2013). *Pir* genes of the S7 clade within particular loci called acute-associated loci (AAPLs) are more highly expressed in the acute-phase of infection and are associated with avirulent infection, whereas a more restricted number of *pirs* from the L1 clade that are associated with the chronic-associated loci (ChAPLs) are highly transcribed during the chronic-phase of infection and are associated with a more virulent course of infection (Brugat et al., 2017). This change from predominantly S7 to L1-expressing parasites and this association has suggested a role of *pirs* in virulence of the blood-stage infections (Brugat et al., 2017).

Earlier immunofluorescence studies using *P. chabaudi pir* genes expressed in *Plasmodium yoelii*, or transgenic *P. berghei* parasites expressing a known *pir*/*bir* gene and *P. falciparum* expressing a *Plasmodium vivax pir*, have shown PIR proteins to be present in all compartments of an iRBC during asexual development: exported to the host-cell cytosol, on the parasitophorous vacuolar membrane (PVM) or within the parasitophorous vacuole (PV) and parasite cytosol (Cunningham et al., 2005; Del Portillo et al., 2001; Fougere et al., 2016; Pasini et al., 2013; Yam et al., 2016) and are also associated with lipid rafts (or detergent-resistant membranes) in these species (Di Girolamo et al., 2008). Some studies indicate that, in fixed preparations of *Plasmodium*-infected RBCs, PIR proteins may also be present on, or close to the infected erythrocyte surface (Cunningham et al., 2005; Del Portillo et al., 2001; Pasini et al., 2013; Yam et al., 2016). A number of other *P. falciparum* multigene families, known to be associated with the parasites’ ability to sequester and evade the host immunity, are also destined for the surface of iRBC, through the Maurer’s clefts, the parasites’ trafficking platforms for export of proteins to the erythrocyte surface (Sam-Yellowe 2009). The foremost exported protein *via* the Maurer’s clefts is *Pf*EMP1, also a virulence factor that mediates cytoadherence and sequestration of late-stage–infected erythrocytes in deep tissues (Baruch et al., 1996; Kyes et al., 2001; Leech et al., 1984; Su et al., 1995). Other virulence proteins transiently associated with Maurer’s clefts on their way to the erythrocyte plasma membrane are the *P. falciparum* RIFINs, STEVOR, *Pf*MC-2TMm, FIKK kinase and probably members of the CLAG family (Ekland et al., 2011; Kaviratne et al., 2002; Nunes et al., 2007; Przyborski et al., 2005; Tsarukyanova et al., 2009).

Here, we show that neither S7 nor L1 PIR proteins could be detected on the surface of live unfixed iRBC, suggesting that they are unlikely to be targets of surface variant-specific antibodies or be involved directly in adhesion of iRBC to host cells. The differences in subcellular localisation of the two major clades of *P. chabaudi* PIRs across the blood cycle and the apparent lack of expression on the red cell surface, strongly suggest that this gene family possibly has diverse functions that could further differ from those known functions of the *var* genes in *Plasmodium falciparum.* Using the *P. chabaudi* model for PIRs, we report here a detailed study of PIR expression of the two major clades of *P. chabaudi* PIR proteins, S7 and L1, in blood-stage parasites following mosquito transmission, in order to obtain a clearer definition of their location in RBC infected with wild-type (WT) *P. chabaudi* parasites. Elucidation of their localisation and proteomic profile following natural transmission may give us clues to the potential functions of PIR proteins and cell-cell interactions during malaria infection.

## 2 Materials and Methods

### 2.1 Mouse and Parasite Lines Used

All experiments were performed in accordance with the UK Animals (Scientific Procedures) Act 1986 (PPL 70/8326 and PADD88D48) and were approved by The Francis Crick Institute Ethical Committee. V(D)J recombination activation gene RAG1 knockout (RAG1^-/-^) (Mombaerts et al., 1992) on a C57BL/6 background and C57BL/6 WT mice were obtained from the specific-pathogen-free (SPF) unit and subsequently conventionally housed with autoclaved cages, bedding and food at the Biological Research Facility (BRF) of the Francis Crick Institute. Experiments were performed with six- to eight-week-old female mice under reverse light conditions (light 19:00pm-07:00am and dark 07:00am-19:00pm), at 20-22°C.

Cloned lines of *P. chabaudi chabaudi AS* parasites were used (originally obtained from David Walliker, University of Edinburgh, UK). To initiate infection, mice were injected intraperitoneally (i.p.) with 1 x 10^5^ infected erythrocytes or submitted to the bites of 25 infected mosquitoes, as previously described (Spence et al., 2012). Parasitaemia was monitored by the counting of blood stage parasites in air dried, methanol fixed, and Giemsa (Sigma)-stained thin tail-blood smears.

### 2.2 Production of *P. chabaudi* Anti-Peptide Antisera

**Figure 1:**
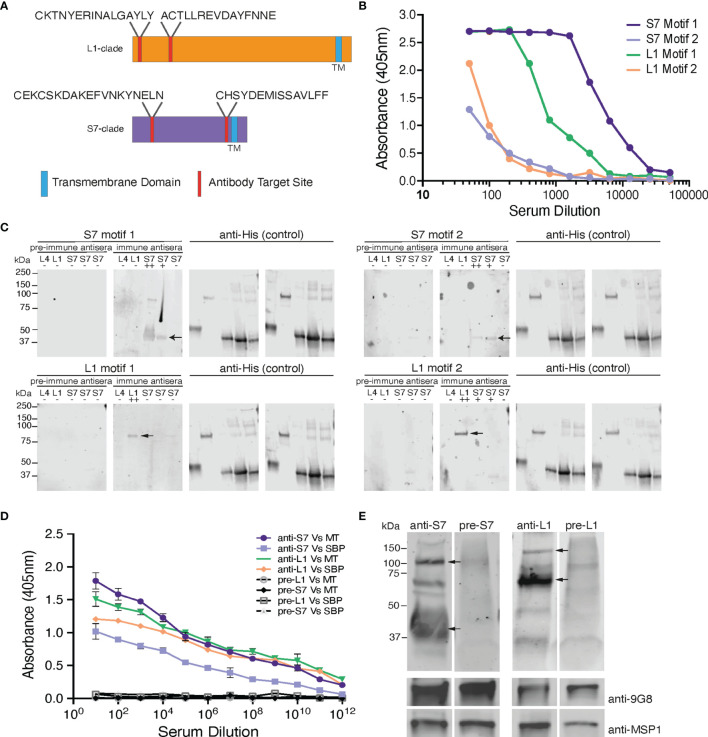
Design and validation of the anti-S7 and anti-L1 peptide antisera. **(A)** Schematic model of S7- and L1-clade proteins showing position of the designed target peptide motifs. One of the S7 target motifs is located towards the N-terminus of the S7 protein clade, while the other is located further towards the C-terminus. Both the target motifs identified in the L1 PIRs are located towards the N-terminus of the protein sequence. TM, Transmembrane Domain. **(B)** ELISA reactivity of the individual anti-S7 and anti-L1 peptide sera (anti-S7 motif 1 peptide antiserum, anti-S7 motif 2 peptide antiserum, anti-L1 motif 1 peptide antiserum, anti-L1 motif 2 peptide antiserum) against the target *P. chabaudi* PIR peptides that were used to immunise rabbits. ELISAs performed by Cambridge Research Biomedicals, and figure adapted from those supplied by Cambridge Research Biochemicals. **(C)** Western blot analysis of S7, L1 and L4 his-tagged recombinant proteins probed with the individual anti-S7 and anti-L1 peptide antisera. Migration of S7 proteins was between 33-37 kDa markers and of L1 proteins between the 75-150 kDa markers (arrows indicate monomers). Probing against the his-tag served as loading control. Pre-immune sera served as negative controls, where they were found to be unreactive and did not recognise any of the recombinant proteins, confirming the lack of pre-existing reactivity to PIRs in the rabbit serum. ++ denotes a protein that highly matches the sequence of the peptide motif that the sera were raised against; + denotes a protein that contains a similar peptide motif (50-60% alignment) to that used for raising the immune sera; - denotes a protein that it does not contain any of the anti-sera target motifs. The specific recombinant proteins loaded are the PCHAS_0600600 (L4), PCHAS_1100300 (L1), PCHAS_0400300 (S7), PCHAS_1200500 (S7) and PCHAS_1300101 (S7). **(D)** ELISA reactivity of the pooled anti-S7 and anti-L1 peptide sera (pooled motif 1 and 2 for each antiserum) against WT parasite lysates extracted from MT and SBP acute-stage parasites. Pre-immune serum served as negative control. OD 405 ± standard deviation of three replicate assays is shown. MT, Mosquito Transmitted; SBP, Serially Blood Passaged. **(E)** Western blot analysis of whole parasite lysates (reducing conditions) prepared from *P. chabaudi* MT WT parasites at the trophozoite-stage, using the anti-S7 and anti-L1 pooled peptide immune sera. Arrows indicate the estimated average size of the monomers or dimers of each PIR clade (S7 33-50 and 100 kDa; L1 75-150 kDa). Probing with the pre-immune sera served as negative control. Probing against the *Plasmodium* EXP2 was used as stage-specific and loading control. The molecular weights on the right indicate the positions to which size markers had migrated.

Protein sequences from all members of the S7 and L1 PIR clades were collated and analysed *via* the MEME suite of bioinformatics software to identify the top ten most conserved peptide motifs within those clades. From these motifs, those located within the C-terminal hydrophobic domain of the proteins were excluded from further analysis. The remaining motif sequences were aligned to their target and off-target PIR clades, to choose those that match as many proteins as possible within the target clade and *vice versa*. The original sequences, taken directly from the candidate PIR proteins, were taken forward. L1 motif 1: CKTNYERINALGAYLY; L1 motif 2: ACTLLREVDAYFNNE; S7 motif 1: CEKCSKDAKEFVNKYNELN; S7 motif 2: CHSYDEMISSAVLFF. Both L1 motifs match the N-terminal of the L1 clade PIRs (39% and 76% conserved among all L1-clade members, respectively), whereas one of the S7 motifs aligns at the N-terminal (58% conserved among all S7-clade members) and the other at the C-terminal (45% conserved among all S7-clade members) of the S7 clade PIRs (**Figure 1A**). The proportion of L1 and S7 clade PIRs targeted by each peptide are summarised in **Supplementary Table S1**. BlastP hits and scores are listed in **Supplementary Table S2**.

The peptides were commercially generated and chemically coupled to a KLH carried protein in order to stabilize the peptide and elicit a stronger and more persistent immunological response. The conjugated to KLH peptides were then used to immunise rabbits and generate the anti-S7 and anti-L1 specific antisera (Cambridge Research Biochemicals, Billingham, UK). Their immunoreactivity to the peptides was verified using standard Enzyme-Linked Immunosorbent Assay (ELISA) procedures. To maximise the proportion of PIRs belonging to the same clade that are recognised by the individual peptide antisera (named anti-S7 motif 1 peptide antiserum, anti-S7 motif 2 peptide antiserum etc), we pooled together the two antisera generated against the two individual motifs that belong to each clade (named anti-S7 and anti-L1 peptide antisera). The individual peptide antisera were mixed proportionally to guarantee similar levels of reactivity with the PIR proteins.

### 2.3 *P. chabaudi* PIR Proteins Expression and Purification

Synthetic genes encoding a panel of *P. chabaudi* PIR proteins (PCHAS_1200500, PCHAS_1100300, PCHAS_0600600, PCHAS_0400300 and PCHAS_1300101) were cloned into the AscI (NEB) and NotI (NEB) restriction sites of the pTT3 vector (Crosnier et al., 2013) that carries a C-terminal His6 tag. Putative C-terminal hydrophobic domains were removed, and putative *N*-linked glycosylation sites were removed through mutation of the S or T residue of the NxS/T motif to alanine.

Protein expression and purification were carried out as previously described (Harrison et al., 2020). Briefly, all plasmids were transfected into HEK293F cells (Thermo Fisher Scientific) using polyethylenimine, and after five days (density of about 2.5x10^6^ cells/mL) harvested by centrifugation at 5,000 × *g*. The supernatant was buffer-exchanged into 20 mM HEPES pH 7.5, 150 mM NaCl, and 20 mM imidazole by tangential flow filtration, and the protein was purified by immobilised metal affinity chromatography using Ni^2+^-NTA resin, followed by size exclusion chromatography using a Superdex 75 10/300 column (GE Healthcare Life Sciences).

### 2.4 ELISA

PCHAS_1200500, PCHAS_1100300, PCHAS_0600600, PCHAS_0400300 and PCHAS_1300101 were purified as described above and re-solubilised in 1 x PBS. For the preparation of parasite protein lysates, whole blood was depleted of leukocytes by filtration (Plasmodipur, EuroProxima) and red cell debris by saponin lysis (Sigma) and centrifugation. Purified parasites were then lysed in RIPA buffer (Sigma) supplemented with 1% SDS (ThermoFisher) and 10 μg/mL (1X) Protease Inhibitors (PI) (cOmplete EDTA free, Roche). Approximately 5 μg/mL in 1 x Phosphate Buffered Saline (PBS) (pH 7.4) of each recombinant protein and 50 μg/mL in 1 x PBS of parasite protein lysate were propped with the titrated (two-fold serial dilutions) rabbit anti-peptide antisera, using standard ELISA assay procedures (Spence et al., 2013). Starting concentrations of the antisera were anti-S7 motif 1 peptide antiserum (1:500), anti-S7 motif 2 peptide antiserum (1:500), anti-L1 motif 1 peptide antiserum (1:500), anti-L1 motif 2 peptide antiserum (1:500), anti-S7 peptide antiserum (pooled motif 1 and 2) (1:1000), anti-L1 peptide antiserum (pooled motif 1 and 2) (1:1000), anti-S7 pre-immune serum (1:1000) and anti-L1 pre-immune serum (1:1000) in 1% (w/v) BSA, 0.3% (v/v) in 1x PBS, incubated at 37°C for 1.5 h. Following three washes with 0.25% (v/v) Tween-20 (Sigma) in 1 x PBS, wells were incubated with goat anti-rabbit IgG (H+L) alkaline phosphatase conjugated secondary antibody (Southern Biotech) for 1 h at 37°C. Samples were developed with 1 mg/mL 4-Nitrophenyl phosphate disodium salt hexahydrate (PNPP; Sigma) and OD was measured at 405 nm on a Tecan Infinite M1000 plate reader (LifeSciences). Data were analysed with Magenta software (LifeSciences).

### 2.5 Membrane Fractionation of Parasitised Erythrocytes, Protein Sample Preparation and Western Blotting

For the preparation of *P. chabaudi* membrane fractionated samples, magnetically enriched infected erythrocytes at the trophozoite-stage were pelleted in aliquots of 2.5 x 10^6^ iRBC and then resuspended into approximately 20 μL (10 volumes of the pellet volume) in 1 x PBS containing 4 haemolytic units of activated Streptolysin-O (SLO) (Sigma), as previously described (Jackson et al., 2007; Ruecker et al., 2012). Following incubation for 15 min at 37°C, the parasite-containing fractions were separated by centrifugation at 12,400 × g for 10 min at 4°C. Parasite fractions were then washed twice in ice cold 1 x PBS containing 10 μg/mL (1X) PI and resuspended in 10 volumes of 0.15% (w/v) saponin (Sigma) in 1 x PBS for 10 min at 4°C. Following centrifugation, pellets were washed twice in ice cold 1 x PBS/PI and resuspended in 50 mM Tris HCl-PBS-PI (pH 7.4) buffer, mechanically homogenised for 1 min and stored at -80°C until frozen. Immediately upon thawing, the samples were centrifuged and washed twice in ice cold 1 x PBS/PI. The remaining membrane soluble fractions were extracted in RIPA whole cell lysis buffer supplemented with 1% SDS and PI for 20 min at 4°C. Following centrifugation, Laemmli buffer (BioRad) was added to all samples used for western blot analysis, or directly stored at -80°C until further proteomic analysis. Whole protein lysates were prepared as described above. After the last centrifugation, the supernatant was mixed with Laemmli buffer until further western blot analysis. Approximately 70 ng of purified recombinant proteins were mixed with Laemmli buffer until further western blot analysis. For reducing conditions, +3-5% (v/v) 2-mercapthoethanol was added to the sample buffer.

Proteins from all samples were separated on a 4–20% gradient polyacrylamide gel electrophoresis (Mini-PROTEAN TGX™ Precast Protein Gels, BioRad) in Tris-Glycine buffer (BioRad), followed by transfer to nitrocellulose membranes (iBlot Transfer Stack, Invitrogen). The blots were then blocked in 3% (w/v) BSA in 1 x PBS (blocking buffer) and probed with the following antibodies: rabbit anti-S7 motif 1 peptide antiserum (1:1000), rabbit anti-S7 motif 2 peptide antiserum (1:1000), rabbit anti-L1 motif 1 peptide antiserum (1:1000), rabbit anti-L1 motif 2 peptide antiserum (1:1000), rabbit anti-S7 peptide antiserum (pooled motif 1 and 2) (1:1000), rabbit anti-L1 peptide antiserum (pooled motif 1 and 2) (1:1000), rabbit anti-S7 pre-immune serum (1:1000), rabbit anti-L1 pre-immune serum (1:1000), mouse anti-his (1:1000; Novagen), mouse anti-9G8 (1:5000), mouse anti-MSP1 (1:2000), rat anti-Ter119 (1:5000), rabbit anti-*Py*EXP2 (a kind gift of James Burns, Drexel University College of Medicine, Philadelphia, United States; Meibalan et al., 2015) (1:500) overnight at 4°C. After three washes with 0.05% (v/v) Tween-20 in 1 x PBS, anti-rabbit IgG 680LT IRDye, anti-mouse 800CW IRDye and anti-rat 800CW IRDye secondary antibody (Li-Cor), diluted 1:10000 in blocking buffer, was applied for 1 h at room temperature. Odyssey Imaging system (Li-Cor) was used for detection and image analysis.

### 2.6 Immunofluorescence Assays and Live Cell Imaging

*P. chabaudi* has a synchronous erythrocytic life cycle and therefore blood could be collected at several time points in the 24-hour cycle in order to study the different asexual stages. Thin smears at the ring-, early trophozoite-, mature trophozoite- and late trophozoite/schizont-stages were prepared at 01 h, 16 h, 19 h and 23 h, respectively. For IFAs on *in vitro* maturated schizonts, infected mouse blood was cultured for five hours as previously described (Spence et al., 2011) and cells were then centrifuged, prior to preparing thin smears. Thin smears were fixed in cold acetone:methanol (9:1) for 10 min at -20°C and air-dried. Fixed cells were then re-hydrated in 1 × PBS, washed once in 1 × PBS, blocked in 3% BSA (w/v) in 1 x PBS for 1 h at room temperature and subsequently incubated with primary antibodies for 1 h at room temperature or overnight at 4°C. Rabbit anti-S7 peptide antiserum, rabbit anti-L1 peptide antiserum, mouse anti-9G8 (Hartz et al., 1993), mouse anti-MSP1 (NIMP23; Boyle et al., 1982), rabbit anti-SBP1-C (a kind gift of Tobias Spielmann, Bernhard Nocht Institute for Tropical Medicine, Hamburg, Germany; De Niz et al., 2016) and rat anti-Ter119 antibodies were used at 1:1000, 1:1000, 1:1000, 1:500, 1:100 and 1:1000 dilution in blocking buffer, respectively. After three washes with 1 x PBS, slides were incubated for 1 h at room temperature with secondary antibodies in blocking solution. Secondary antibodies used were goat anti-rabbit Alexa Fluor 568, goat anti-mouse Alexa Fluor 488 and donkey anti-rat Alexa Fluor 647 (Life Technologies) at 1:1000 dilution in blocking solution. Following three washes with 1 x PBS, nuclei were stained with 1 μg/mL−1 4′,6′-diamidine-2′-phenylindole dihydrochloride (DAPI) (Roche) in 1 x PBS for 5 min at room temperature. Slides were mounted with ProLong antifade mounting medium (ThermoFisher Scientific) and viewed under a Leica SP5 MP confocal laser-scanning microscope. Images were processed by deconvolution using the Huygens software and visualised using the ImageJ x 38 (NIH) software. In these experiments, Ter119 antibodies were used to define the red cell membrane and not direct light microscopic images.

For live cell imaging, parasites were incubated with the primary antibodies for 30 min at room temperature, washed three times in 1 x PBS, and incubated with the secondary antibodies for 20 min at room temperature. The cells were then viewed immediately under a Leica SP5 MP confocal laser-scanning microscope between slide and coverslip, as described above. After acquisition, contrast and brightness levels were optimised using the Image J x 38 software.

### 2.7 Electron Microscopy

Magnetically enriched infected erythrocytes at the trophozoite-stage were pelleted and fixed with 4% (v/v) paraformaldehyde/0.01% (v/v) glutaraldehyde in 1 x PBS for 30 mins at room temperature, followed by a second fixation step using 4% formaldehyde (v/v)/2.5% (v/v) glutaraldehyde in 0.1 M Sorenson’s phosphate buffer (0.1 M Na_2_HPO_4_/ddH2O, pH 7.4) for 60 min at room temperature.

The cell pellet was then processed using a modified version of the NCMIR protocol (Deerinck et al., 2010). Briefly, the cells were washed in 0.1 M phosphate buffer and post-fixed with 1% reduced osmium (1% w/v OsO4/1.5% w/v K_3_Fe(CN)_6_) for 60 min at 4°C, and then washed in double distilled water (ddH2O). The cells were incubated in 1% thiocarbohydrazide (TCH) for 20 min at room temperature, rinsed in ddH2O and further fixed with 2% (w/v) osmium tetroxide for 30 min at room temperature. The cells were then stained with 1% (w/v) uranyl acetate at 4°C overnight, washed in ddH2O and stained with Walton’s lead aspartate for 30 min at 60°C. The cells were then washed in ddH2O and dehydrated stepwise using serial dilutions of ethanol: 30% (v/v) at room temperature for 5 min, then 50% (v/v), 70% (v/v), 90% (v/v) and twice with 100% for 10 min each. The cells were incubated in propylene oxide (PO) for 10 min. The cells were subsequently infiltrated with 3:1 mixture of PO : Durcupan resin (Sigma) for 45 min at room temperature, followed by 1:1 and 1:3 mixtures for 45 min each at room temperature, then with 100% Durcupan resin for 48 h. The cell pellet was then polymerised in fresh Durcupan resin at 60°C for 48 h. The sample was cut into 70 nm ultrathin sections using an ultramicrotome (UC7, Leica Biosystems UK) and picked up onto copper mesh grids (Agar Scientific). Images were obtained on a 120 kV transmission electron microscope (TEM) (JEM-1400Flash, JEOL) using a sCMOS camera (Matataki Flash, JEOL).

### 2.8 Flow Cytometric Investigation of S7 and L1 PIRs Localisation

Investigation of iRBC surface antigens was performed using a flow cytometry-based assay modified protocol from Elsworth et al., 2014. Two microliters of whole blood from C57Bl/6 RAG1^-/- P^ *. chabaudi* AS infected mice (parasitaemia 15-20%) at the trophozoite-stage, were collected in 20 μL of saline-heparin (25 U/mL, Wockhardt) in U-shaped 96-well plates. After two washes with 1 x PBS supplemented with 2% (v/v) foetal bovine serum (FBS) and 3 mM EDTA (ThermoFisher), cells were either stored at 4°C to be stained later or fixed in 500 μL of 1:1 acetone:methanol for 10 mins at -20°C. Fixed cells were then washed twice in 1 x PBS/FBS/EDTA. Both live and fixed cells were then incubated with the anti-S7 and anti-L1 rabbit peptide antisera diluted in 1 x PBS/FBS/EDTA for 45 min at 4°C. Equal amounts of the anti-S7 motifs 1 and 2 and anti-L1 motifs 1 and 2 were mixed together prior to cell staining, and diluted 1:500 in 1 x PBS/FBC/EDTA. Following three washes in 1 x PBS/FBS/EDTA, cells were stained simultaneously with anti-Ter119 PE conjugated antibody (BioLegend) and goat anti-rabbit Alexa Fluor 568 (Life Technologies) for 20 mins at 4°C in the dark. Both the rat anti-Ter119 PE and the goat anti-rabbit Alexa Fluor 568 antibodies were diluted 1/1,000 in 1 x PBS/FBS/EDTA. Cells were then washed twice and stained immediately in 200 μL Hoechst 33342 (10 μg/mL in 1 x PBS/FBS/EDTA) for 15 mins at 37°C to stain for DNA. After washing twice in 1 x PBS/FBS/EDTA, cells were resuspended in 250 μL 1 x PBS/FBS/EDTA and acquired by flow cytometry on an Amnis^®^ CellStream^®^ benchtop flow cytometer (Luminex).

A total of 500,000 single RBC (selected based on their FCS/SSC and FSC/AspectRatio FSC) were analysed for each sample. The gating strategy established for the analysis of the results is described in (**Supplementary Figure S1**). FlowJo (Tree Star) was used for all flow cytometry analyses. Fixation in acetone:methanol also permeabilises the cells, therefore fixed cells were used as a control to validate antibody integrity. “Fluorescence minus one” (FMO) and single staining controls were included as controls, as well as a negative control sample from a non-infected mouse to define the threshold of positivity for the antibody signal.

### 2.9 Mass-Spectrometric (MS) Analyses

#### 2.9.1 MS SDS-PAGE

Approximately 50 μg of the SLO fraction, and 15-20 μg of the SAP, Tris and RIPA fractions were added to 8 μL of Laemmli buffer containing 3% (v/v) 1,4-dithiothreitol (DTT; Roche). The mixture was heated for 10 min at 95°C and ran on a 16.5% Tris-Tricine gel electrophoresis (Mini-Protean Tris-Tricine Precast Protein Gels, BioRad) in Tris-Tricine buffer (BioRad). Gel was allowed to run at constant voltage (120 V) until track lengths (top to tracking dye) averaged 5 cm. Following gel incubation with colloidal Coomassie (abcam) and de-staining in HPLC grade water, protein bands below 17 kDa were excised and discarded to ensure mouse haemoglobin depletion (Mouse haemoglobin size: 14.4 kDa).

#### 2.9.2 In-Gel Digestion and Fractionation

Gel lanes corresponding to each sample were cut into pieces and spread out into 8 wells of a 96-well U-shaped-bottom polystyrene plate (Costar). Gel pieces were de-stained overnight in 50% (v/v) Acetonitrile/100 mM Ammonium Bicarbonate, de-hydrated in 100% Acetonitrile (5 min) and aired-dried for 10 min at room temperature. Following reduction with 10 mM Dithiothreitol (60 min, room temperature) and alkylation with 55 mM Iodoacetamide (30 min, room temperature, in the dark), gel pieces were washed once with 50% (v/v) Acetonitrile/100 mM Ammonium Bicarbonate with shaking (10 min), de-hydrated with 100% Acetonitrile (5 min) and air-dried for 10 min at room temperature.

MS-grade Trypsin (Pierce) in 50 mM Ammonium Bicarbonate was added to each well (200 ng) in sufficient volume to allow for complete gel saturation. Samples were then digested overnight at 37°C with shaking. Peptides were extracted by incubation with 50% (v/v) Acetonitrile/100 mM Ammonium Bicarbonate (20 min) followed by 100% Acetonitrile (10 min). Peptide solutions corresponding to each sample were then pooled and partially dried in a vacuum centrifuge. Quantitative Colorimetric Peptide Assay (Pierce) was used to assess peptide content in each sample. 5 μg aliquots were then removed, acidified with neat Trifluoroacetic acid (TFA) and fractionated on a stage tip using high pH reverse phase (bRP). Briefly, each stage tip was packed with two C18 Empore discs (3M), conditioned with 100 μL of 100% Acetonitrile, followed by 200 µL of 0.1% (v/v) TFA. The samples were loaded in 1% (v/v) TFA, washed once with 200 μL water and fractionated by elution with increasing amounts of Acetonitrile (10% v/v, 17.5% v/v, 25% v/v and 50% v/v) in 0.1% (v/v) Triethylamine. Resulting fractions (25 μL each) were pooled non-consecutively, i.e. fraction 1 with 3 and fraction 2 with 4. The two final bRP fractions were vacuum dried.

#### 2.9.3 LC-MS/MS

Samples were resuspended in 2% (v/v) Acetonitrile/0.1% (v/v) TFA supplemented with iRT standards (Biognosys) and loaded on a 50 cm Easy Spray PepMap column (75 μm inner diameter, 2 μm particle size, Thermo Fisher Scientific) equipped with an integrated electrospray emitter. Reverse phase chromatography was performed using the RSLC nano U3000 (Thermo Fisher Scientific) with a binary buffer system (solvent A: 0.1% v/v Formic acid, 5% v/v DMSO; solvent B: 80% v/v Acetonitrile, 0.1% v/v Formic acid, 5% v/v DMSO) at a flow rate of 275 nL/min. The samples were run on a linear gradient of 2-8% B in 5.5 min, 8-25%B in 54.5 min, 25-40% B in 31 min, and 40-50% B in 7 min with a total run time of 120 min including column conditioning. The nanoLC was coupled to an Orbitrap Eclipse mass spectrometer using an EasySpray nano source (Thermo Fisher Scientific). The Orbitrap Eclipse was operated in data-independent mode (DIA) with following settings: MS1 data acquired in the Orbitrap with a resolution of 120,000, max injection time of 20 ms, AGC target of 1e6, in positive ion mode and profile mode, over the mass range 375-1,500 m/z. DIA segments over this mass range (variable size windows, 34 in total) were acquired in the Orbitrap following fragmentation in the HCD cell (30%), with 30,000 resolution over the mass range 200-2000 m/z and with a max injection time of 70 ms and AGC target of 1e6.

#### 2.9.4 Data Analysis

Acquired raw data were analysed with Spectronaut v15.4.210913 (Biognosys AG) using directDIA analysis option. Data were searched against *P. c. chabaudi* (PlasmoDB), *Mus musculus* (UniProt) and contaminants (Spectronaut) FASTA files using Pulsar search engine. Cysteine carbamidomethylation was selected as a fixed modification, whereas oxidation (M), acetylation (Protein N-term) and deamidation (NQ) were selected as variable modifications. The enzyme specificity was set to trypsin with a maximum of 2 missed cleavages. The datasets were filtered on posterior error probability (PEP) to achieve a 1% false discovery rate on protein, peptide and PSM level. MS1 and MS2 tolerances were set to dynamic and retention time calibration was based on iRT regression generated in Spectronaut. Interference correction was activated, keeping a minimum of 2 precursor ions and 3 fragment ions. Quant 2.0 was selected as protein LFQ method and Quantity MS-level was set to MS2 with Q value ≤ 0.01 used for data filtering. No intensity normalisation was performed.

Data were further processed with Microsoft Office Excel 2016 (**Supplementary Table S3**). Protein group data were filtered to remove common contaminant entries as well as for the presence of 1 valid value. Quantity values were then log2 transformed and no further data transformations were performed. The mass spectrometry proteomics data have been deposited on the ProteomeXchange Consortium *via* the PRIDE (Perez-Riverol et al., 2019) partner repository with the dataset identifier PXD031586.

### 2.10 Statistical Analysis

Statistical analysis was determined by using Graphpad Prism5 software. The one-way analysis of variance (ANOVA) test was used. *P*-value of <0.05 was considered as statistically significant.

## 3 Results

### 3.1 Generation and Validation of Rabbit Antisera

The two largest clades of *P. chabaudi* PIRs, the S7s and the L1s, together account for 68% of the PIRs in *P. chabaudi*. There are 70 S7s and 82 L1s PIRs in the *P. chabaudi* genome. PIR members of each clade share conserved regions (Brugat et al., 2017) and in order to examine their expression and localisation more holistically, we generated peptide antisera that targeted as many members of each PIR clade as possible.

The localisation of PIR proteins within *P. chabaudi*-parasitised erythrocytes during the acute- and chronic-phases of infection was investigated, using rabbit polyclonal antisera that were generated against 20-conserved residue sequences of the S7 (acute-associated) and of L1 (chronic-associated) PIR clades, respectively (Freitas-Junior et al., 2000; Otto et al., 2014), the two major *pir* clades expressed in *P. chabaudi* blood-stage parasites (Brugat et al., 2017; Little et al., 2021). Two peptide motifs were chosen for each clade (**Figure 1A**), whose sequence had the highest number of BlastP hits within the same clade they were designed for and the lowest off-target activity. In summary, the peptides recognised by the anti-S7 peptide antisera designed against motif 1 and motif 2 are present in 37/70 and 12/70 S7 PIRs, respectively. Similarly, the peptide motif 1 and motif 2 identified for the L1 clade can be found in 49/82 and 29/82 of the L1 PIRs, respectively. Some off-target proteins (i.e. PIRs of a different clade) could not be avoided. Peptide sequences are described in **Supplementary Table S1**, and materials and methods. The BlastP hits for each motif designed against each clade are summarised in **Supplementary Table S2**.

Antisera to the selected peptides, generated in rabbits (Cambridge Research Biochemicals, Billingham, UK) were tested by ELISA against their respective target peptides (**Figure 1B**). Peptide antisera that were generated against S7 motif 1 and L1 motif 1 recognise their target peptide at high titres, whereas peptide antisera against the S7 motif 2 and L1 motif 2 antigens indicated lower titres. Western blot analysis against the his-tagged recombinant PIR proteins PCHAS_0600600 (L4), PCHAS_1100300 (L1), PCHAS_0400300 (S7), PCHAS_1200500 (S7) and PCHAS_1300101 (S7) confirmed specificity of each antiserum to their target clades (S7 at approximately 33-37 kDa, L1 at approximately 75-151 kDa, and L4 at approximately 40 kDa). Pre-immune rabbit antisera did not recognise any of the recombinant proteins (**Figure 1C**).

To maximise the proportion of PIRs belonging to the same clade that are picked up by the individual peptide antisera, we pooled together the two antisera generated against the individual motifs from each clade. The pooled antisera were then checked for reactivity against the PIR proteins in *P. chabaudi* WT parasites by ELISA and western blot analysis. ELISA assays using a lysate of *P. chabaudi* blood-stage parasites showed that both rabbit anti-S7 and anti-L1 sera contained antibodies that recognised proteins from lysates of both MT and serially passaged blood-stage (SBP) parasites (**Figure 1D**), with the overall reactivity being higher for both antisera with the parasite lysate derived from MT blood-stage parasites. The increased reactivity of the antisera with MT blood-stage parasites agrees with the increased RNA expression in MT parasites compared with SBP parasites, previously observed (Spence et al., 2013; Brugat et al., 2017). Western blot analysis of the pooled rabbit anti-S7 and anti-L1 sera against whole protein lysates of *P. chabaudi WT* MT parasites demonstrated that both antisera are able to recognise naturally produced PIR proteins. The two antisera display differing patterns of bands across the lysates, with S7 and L1 antisera having predominant bands of 33-50 kDa, and 75-151 kDa, respectively, reflecting the expected size of *PIR* proteins of the S7 and L1 clades (**Figure 1E**). The S7 antisera also interact with a protein of approximately 100 kDa, which may be due to formation of multimers, as purified recombinant protein exhibits a similar migration and interaction (**Figure 1C**). Arrows indicate the major interacting proteins.

### 3.2 Localisation of S7 and L1 PIRs in Mature *P. chabaudi* Trophozoites in Acute- and Chronic-Phases of Infection

**Figure 2:**
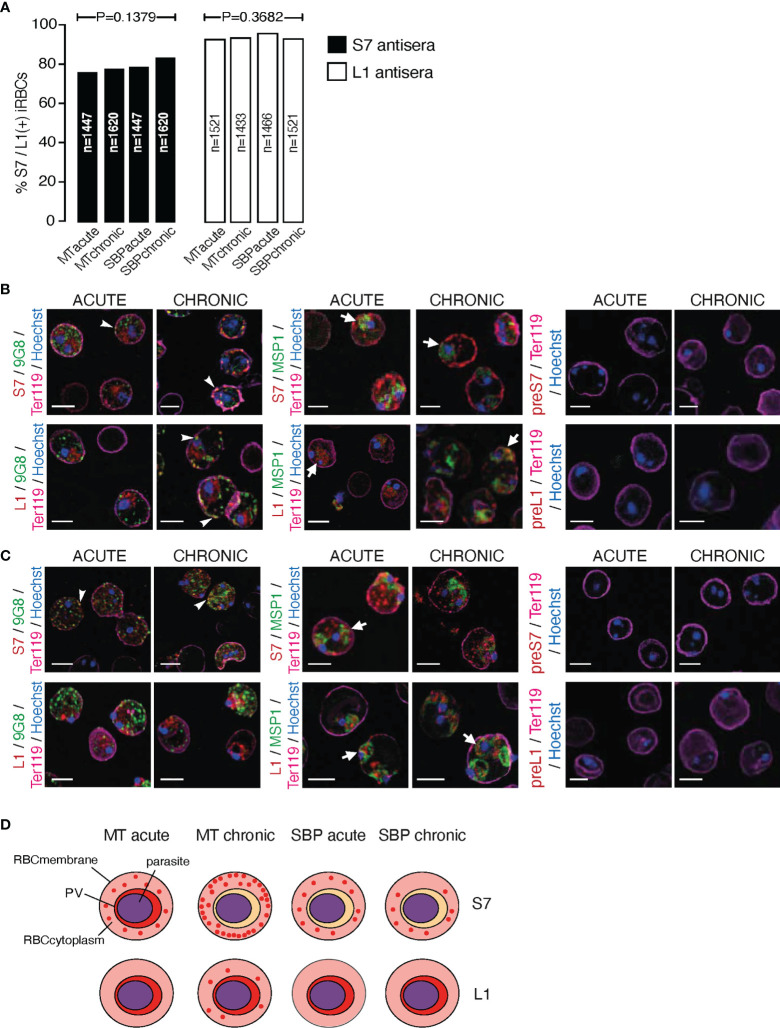
Subcellular localisation of S7 and L1 PIRs in the late trophozoite-stage of MT and SBP *P. c. chabaudi AS* parasites, during the acute- and chronic-phases of infection. **(A)** Percentage of MT and SBP S7-positive (dark grey) and L1-positive (light grey) parasitised erythrocytes, at the late trophozoite-stage. N represents the total number of iRBC analysed in three biological replicates. Data are presented as mean with SEM. Statistical significance was calculated using one-way Analysis Of Variance (ANOVA) tests (P < 0.05). **(B, C)** Immunofluorescence assays of MT **(B)** and SBP **(C)** WT *P. chabaudi* parasites at the late trophozoite-stage. Blood was isolated from C57Bl/6 RAG1^-/-^ mice. Parasites were primarily stained with the anti-S7 or anti-L1 peptide sera (red), and later with the anti-clone 6 (9G8) or anti-MSP1 monoclonal antibodies (green). The white arrowheads indicate co-localisation of the S7s or L1s with 9G8, whereas the white arrows indicate co-localisation of the S7s and L1s with MSP1. Erythrocyte membrane was stained with the rat anti-Ter119 monoclonal antibody (magenta), and parasite nuclei were stained with Hoechst (blue). Staining with pre-immune sera was used as negative control (right panel). Images were taken from confocal sections of acetone:methanol fixed parasites at X630 magnification. Scale bar length corresponds to 5 μm. **(D)** Schematic representation summarising the results obtained following confocal imaging of MT and SBP *P. chabaudi* parasites. The localisation pattern of S7s and L1s across the acute- and chronic-phases of infection in MT and SBP parasites is indicated in red. RBCmembrane, Red Blood Cell Membrane; PV, Parasitophorous Vacuole.

The proportion of acetone:methanol fixed/permeabilised *P. chabaudi* iRBC that stained positively for S7 and L1 PIRs is similar between acute and chronic blood-stage infection and between MT and SBP parasites (**Figure 2A**). However, the proportion of L1-positive iRBC (mean value: 95.33 ± 0.53% SEM) is significantly higher (p=0.0286) in all cases, compared to the percentage of positive cells enumerated for the anti-S7 serum (mean value: 78.95 ± 0.60% SEM). Individual *P. chabaudi* parasites are thought to co-express multiple *pir* genes, as well as expressing many *pir* genes at a population level (Bozdech et al., 2008; Cunningham et al., 2009; Yam et al., 2016). Our data suggest that at each stage, individual parasites also express multiple PIR proteins across different clades.

Subcellular localisation of S7 and L1 PIRs was investigated in fixed/permeabilised RBC from *P. chabaudi* infections, at both acute- and chronic-phases of the infection (at day 10 and day 35 post infection, respectively) (Brugat et al., 2017) in immunofluorescence assays using the anti-S7 and anti-L1 peptide antisera described above. Trophozoite-stage parasites from MT blood-stage *P. chabaudi* were initially assessed, as it has been previously demonstrated that *pir* transcripts are increased in blood-stage parasites following mosquito transmission (Spence et al., 2013; Yam et al., 2016; Brugat et al., 2017). In iRBC taken from an acute-stage MT infection, S7 PIRs are mainly exported into the host’s iRBC cytoplasm where they can be seen as multiple discrete foci. They are also seen, less abundantly, close to the PVM. This pattern changes in iRBC collected at the chronic phase of infection, where in approximately 98% of iRBC, PIRs were localised closed to the iRBC surface and to a lesser extent exported into its cytoplasm (**Figure 2B**; top panel, **Supplementary Figure S2**). L1 PIRs in acute-phase MT blood-stage parasites are localised on or close to the PVM, but with progression to the chronic-phase of infection, they also translocate to the iRBC cytoplasm. Specifically, in approximately 80% of the iRBC, L1 PIRs persist in close proximity to the PVM while also being secreted within the iRBC cytoplasm where they accumulate in granular structures that further co-localise with clone 6 following labelling with 9G8, a monoclonal antibody that recognises a tryptophan-rich protein (PCHAS_0624800) known to be found in the cytoplasm of *P. chabaudi* iRBC (Hartz et al., 1993) (**Figure 2B**; bottom panel, **Supplementary Figure S2**).

Transcription of *pir* genes in MT *P. chabaudi* iRBC differs from that observed in the more commonly used SBP parasites, in both amount and diversity of *pir* genes expressed (Spence et al., 2013; Brugat et al., 2017). This seems to also apply at the expression level where PIR proteins are more highly abundant in MT parasites compared to SBP, as described above (**Figure 1D**). We therefore asked whether PIRs in *P. chabaudi* SBP infected erythrocytes also have a different localisation pattern compared to the MT parasitised erythrocytes. Immunofluorescence staining of *P. chabaudi* SBP iRBC at the acute- and chronic-phases of infection with the anti-S7 and anti-L1 peptide antisera, have shown that S7 PIRs are exported into the iRBC cytoplasm during both the acute- and chronic-phases of a SBP infection (**Figure 2C**; top panel), whereas L1 PIRs are localised in close proximity to the PVM in both acute- and chronic-phases parasites (**Figure 2C**; bottom panel).

Neither S7 nor L1 PIR proteins could be detected at the surface membrane of RBC infected with either MT or SBP blood-stage parasites (**Figures 2B, C** **)**. However, as there are predicted transmembrane domains in PIR proteins (Harrison et al., 2020), S7s may be located on vesicle-like structures within the iRBC cytoplasm and L1s on the PVM. In agreement with this, S7 showed co-localisation with 9G8 monoclonal antibodies, and L1 was found to be localised closely to MSP1, known to be concentrated at the parasite periphery which is consistent with PV localisation (Holder et al., 1992). Some co-localisation of the S7 PIRs with MSP1 was observed in acute-phase MT blood-stage parasites, whereas L1 PIRs seem to translocate on a similar vesicle-like pattern as 9G8 when progressing into the chronic-phase of MT blood-stage parasites. For clarification, the localisation data are summarised in a schematic (**Figure 2D**).

### 3.3 Localisation of S7 and L1 PIRs Throughout the Asexual Developmental Cycle

**Figure 3:**
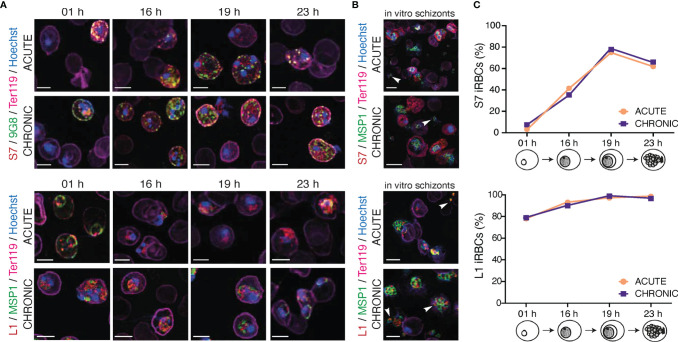
Localisation pattern of S7 and L1 PIRs across the 24-hour asexual blood cycle of MT *P. c. chabaudi AS* parasites, during the acute- and chronic-phases of infection. **(A, B)** Immunofluorescence assays of MT WT *P. chabaudi* parasites at different developmental stages of their asexual blood cycle. WT mice were directly exposed to infected mosquitoes and following infection establishment, acute- (at day-7 post infection) or chronic-phase (at day-45 post infection) parasites were passaged into C57Bl/6 RAG1^-/-^ mice. Upon infection establishment, blood tail smears were prepared for immunofluorescent staining at the ring (1 h), early trophozoite (16 h), mature trophozoite (19 h) **(A)**, and late trophozoite/schizont (23 h) **(B)** developmental stages. Blood smears from *in vitro* schizont cultures, following five hours of culture, were also prepared. Parasites were primarily stained with the anti-S7 (top panels) or anti-L1 peptide sera (bottom panels) (red). S7-incubated slides were then probed with the anti-clone 6 (9G8) (green) antibody, whereas L1-incubated slides were probed with the anti-MSP1 monoclonal antibody (green). The RBC surface membrane was stained with an anti-Ter119 antibody (magenta), and parasite nuclei were stained with Hoechst (blue). Images were taken from confocal sections of acetone:methanol fixed parasites at X630 magnification. Asterisks indicate free merozoites. Scale bar length correspond to 5 μm. **(C)** Percentage of S7-positive (top panel) and L1-positive (bottom panel) iRBC throughout the 24-hour life cycle, in MT parasites during acute- and chronic-phases of infection. About 950-1,000 red blood cells were analysed across two individual biological replicates, where data are presented as mean.

PIR expression in iRBC during acute and chronic *P. chabaudi* infection described above was determined using only the trophozoite-stage of the parasite. We next examined whether the subcellular localisation of the S7 and L1 PIRs changed during the 24-hour developmental cycle in iRBC taken from acute- and chronic-stages of an MT infection using the S7 and L1 peptide-specific antisera. PIR localisation was examined at rings (01 h), early trophozoites (16 h), mature trophozoites (19 h) and late trophozoites/schizonts (23 h) on blood films directly *ex vivo* (**Figure 3A** **;** **Supplementary Figure S3**). However, as *P. chabaudi* schizonts are normally not present or only at very low levels in peripheral blood because of sequestration in organs and tissues (Gilks et al., 1990; Mota et al., 2000; Brugat et al., 2014), PIR expression was also determined in schizont-iRBC that had been matured *in vitro* (**Figure 3B**).

S7 PIRs were exported to the iRBC cytosol and appeared as dense granules, in both trophozoite- and ring-stages of both acute- and chronic-phase parasites. In agreement with our observations for trophozoites described in **Figure 2**, S7 PIRs appeared to be more closely associated with the iRBC membrane in the chronic-phase of infection throughout the 24-hour cycle. Furthermore, co-localisation of the S7 PIRs with the tryptophan-rich protein recognised by the 9G8 antibody indicates vesicle-like distribution of the S7s across the iRBC cytoplasm (**Figure 3A**; top panel). L1 PIRs were found to be associated with the PVM during the ring- and trophozoite-stages; further supported by their co-localisation with the merozoite surface protein MSP1. With progression to the chronic-phase of infection and during ring-to-trophozoite developmental transition, traces of L1 PIRs were seen in the host cell cytoplasm (**Figure 3A**; bottom panel), validating our previous observations.

In *in vitro*-matured schizonts, S7 PIRs were found to be exclusively exported beyond the parasite plasma membrane and PVM, and distributed evenly throughout the iRBC cytosol. However, in the few cases where free merozoites were detected, upon schizont rupture, S7 PIRs were found to be highly associated with their cytoplasm as a single vesicle-like structure; about 80% of the released merozoites were found to be positive for S7 expression. Progression from acute- to chronic-phases of infection did not alter S7 PIR localisation (**Figure 3B**; top panel). L1 PIRs in *in vitro* schizonts from both acute- and chronic-phase were found to be localised within the individual merozoites’ cytoplasm as discrete dots. Upon schizont rupture, L1 expression could still be observed in approximately 95% of the released merozoites possibly suggesting a potential function of the L1s in merozoite egress or invasion. Co-localisation of the L1s with MSP1 was not observed, further suggesting that L1 PIRs are not localised on the merozoites surface (**Figure 3B**; bottom panel). Similar distribution pattern of the S7 and L1 PIRs has been also observed in SBP acute-stage *in vitro* maturated schizonts (**Supplementary Figure S4**).

These results show that localisation of both S7 and L1 clades does not change across *P. chabaudi* 24-hour developmental cycle in the blood. The only difference observed between expression in the different developmental stages was the proportion of iRBC that stained positively with the peptide antisera (**Figure 3C**). The proportion of detectable S7-expressing cells peaked at 19 h (mature trophozoites), after which they rapidly declined with minimum expression levels at 01 h (ring-stage). The proportion of L1-expressing cells did not significantly change between early (16 h) and late trophozoites (19 h), at 97-98% abundance. After the trophozoite-stage, the proportion of L1-positive iRBC decreased to about 77-78% abundance in the ring-stage. Importantly, the proportion of S7+/L1+ iRBC in acute and chronic *P. chabaudi* parasites did not change indicating the lack of further regulation between acute- and chronic-phase.

### 3.4 Maurer’s Clefts in the Cytosol of *P. chabaudi* Infected Erythrocytes Show Similar Localisation Pattern to S7 PIRs

**Figure 4:**
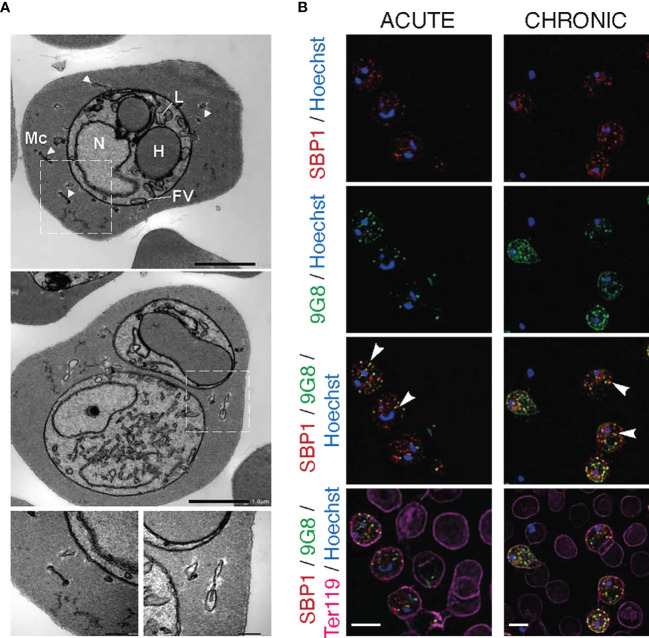
Maurer’s clefts-like structures in *P. c. chabaudi AS* infected erythrocytes. **(A)** Transmission electron micrographs of a *P. chabaudi* MT acute phase trophozoite, inside a mouse erythrocyte. The white arrowheads indicate Maurer’s clefts within the iRBC cytosol. Insets show sections of the areas highlighted. Scale bar length corresponds to 1 μm and 0.2 μm in insets. FV, Food Vacuole; H, Haemoglobin Filled Compartment; L, Lipid Droplets; Mc, Maurer’s clefts; N, Nucleus. **(B)** Immunofluorescence assays of MT acute- (left panels) and chronic- (right panels) phase WT *P. chabaudi* parasites at the late trophozoite-stage. Blood was isolated from C57Bl/6 RAG1^-/-^ mice. Parasites were primarily stained with the anti-PbSBP1 antibody (De Niz et al., 2016) (red) and then with the anti-clone 6 (9G8) monoclonal antibody (green). The RBC surface membrane was stained with the anti-Ter119 monoclonal antibody (magenta), and parasite nuclei were stained with Hoechst (blue). The white arrowheads indicate co-localisation of SBP1 with 9G8. Images were taken from confocal sections of acetone:methanol fixed parasites at X630 magnification. Scale bar length corresponds to 5 μm.

Previous staining of MT acute and chronic *P. chabaudi* blood-stage parasites with the anti-S7 peptide antisera displayed a similar punctuate localisation pattern to that of clone 6 following immunostaining with the 9G8 monoclonal antibody (**Figure 2B**). This vesicle-like pattern of both S7 PIRs and clone 6 is typical of Maurer’s clefts. Maurer’s clefts are sack-like structures spread across the iRBC cytosol of most malaria species (Langreth et al., 1978; Atkinson and Aikawa, 1990; Henrich et al., 2009) and they have been shown to be involved in sorting parasite proteins on the surface of iRBC (Mundwiler-Pachlatko and Beck, 2013). The presence of Maurer’s clefts has not yet been examined in *P. chabaudi*. By conducting electron microscopy (**Figure 4A**) and fluorescence (**Figure 4B**) imaging analysis of *P. chabaudi* iRBC at the trophozoite-stage, we investigated whether Maurer’s clefts exist in the cytoplasm of *P. chabaudi* parasitised erythrocytes, in similar vesicle-like structures as in other malaria species.

**Figure 5:**
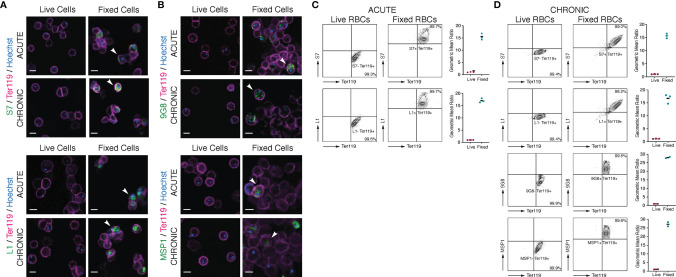
Localisation of S7 and L1 PIRs in live acute and chronic MT *P. c. chabaudi AS* parasitised erythrocytes. **(A, B)** Immunofluorescence staining of live (left panels) and acetone:methanol-fixed iRBC (right panels) of MT *P. chabaudi* parasites at the late trophozoite-stage, isolated from blood of C57Bl/6 RAG1^-/-^ mouse during acute (at day-7 post infection) (upper panels) and chronic (at day-45 post infection) (lower panels) infection. **(A)** *P. chabaudi* parasites were stained with either anti-S7 or anti-L1 peptide antisera (green). **(B)** 9G8 (upper panels) or anti-MSP1 (lower panels) monoclonal antibodies, which recognise parasite antigens internal to the iRBC or parasite, were used as negative controls for live imaging and positive controls for imaging the acetone:methanol fixed iRBC. The RBC surface membrane was stained with anti-Ter119 antibody (magenta), and parasite nuclei were stained with Hoechst (blue). Staining with pre-immune rabbit serum was used as a negative control in the case of peptide antisera (data not shown). The white arrowheads in the panels with fixed iRBC indicate L1, S7, 9G8 or MSP1 positive cells. Images were taken from confocal sections of live and acetone:methanol fixed parasites at X630 magnification and are representative of a total of 500-600 iRBC imaged across two individual replicates. Scale bar length corresponds to 5 μm. **(C, D)** Live *P. chabaudi* iRBC at the acute- **(C)** and chronic- **(D)** phases of infection in C57Bl/6 WT mice were labelled with the anti-S7 or anti-L1 peptide antisera, and anti-Ter119 monoclonal antibody, and analysed using the Amnis^®^ CellStream^®^ Cytometer. Representative contour plots (of 2-3 mice) show the percentage of the iRBC population (Hoechst+/Ter119+) that is either positive or negative for anti-S7 or anti-L1 peptide antisera binding. The quadrant gate was set based on the anti-S7 or anti-L1 background signal detected in the Hoechst- RBC for each condition. Acetone:methanol fixed cells, which are also permeabilised, were used as a control to confirm antibody reactivity with *P. chabaudi* parasites. 9G8 or anti-MSP1 monoclonal antibodies were used as negative controls for live cells, and as a positive control for staining internal parasite proteins in fixed/permeabilised cells. The side plots show the ratio of the geometric mean fluorescence intensity (MFI) detected following binding of the anti-S7 or anti-L1 antibodies on Hoechst+ and Hoechst-, live and fixed (control) RBC. Higher ratios indicate presence and higher abundance of the investigated proteins in Hoechst+ compared to the Hoechst- cells. Ratio of around 1 indicates no difference in the fluorescence intensity of antibody binding (and therefore protein abundance) between the Hoechst+ and Hoechst- RBC. Three biological replicates (i.e., infected mice) were performed for antibody labelling of the S7 or L1 PIRs, whereas two replicates were conducted for antibody labelling with the 9G8 and anti-MSP1 monoclonal antibodies. Data for each individual mouse are presented in (**Supplementary Figure S5**).

Transmission electron microscopy (TEM) analysis revealed membrane structures with sizes ranging from 30 to 150 nm, scattered between the PVM and the erythrocyte membrane (**Figure 4A**). They appear as narrow flattened clefts with either a dense or slightly expanded lucent lumen, suggesting that these vesicles are likely to be Maurer’s clefts. However, to confirm that these vesicle-like structures are Maurer’s clefts, we performed fluorescence microscopy-based imaging of MT blood-stage parasites at the acute- and chronic-phases of the infection following staining with the *P. berghei* anti-SBP1 antiserum (a gift of Tobias Spielmann; De Niz et al., 2016). *Pb*SBP1 is essential for parasite sequestration (De Niz et al., 2016) and it has been shown to co-localise with IBIS1, a marker protein of previously identified Maurer’s clefts-like structures in *P. berghei* (Ingmundson et al., 2012). Since there are very few available antibodies for *P. chabaudi* and *P. berghei* SBP1 (*Pb*SBP1) shares 64% similarity with its *P. chabaudi* orthologue, anti-*Pb*SBP1 was used as a marker for identifying Maurer’s clefts in *P. chabaudi.* Moreover, since staining with the 9G8 monoclonal antibody displays the same localisation pattern as that of the S7 PIRs, both the anti-*Pb*SBP1 antiserum and 9G8 monoclonal antibody were used to co-stain *P. chabaudi* iRBC. Direct co-localisation experiment using the *P. berghei* anti-SBP1 and the *P. chabaudi* anti-S7 peptide antisera simultaneously was not conducted, as both were produced in rabbits. Immunofluorescence assays showed that SBP1 and clone 6 are exported into the cytosol of *P. chabaudi* acute-stage infected erythrocytes where they were found to be co-localised in multiple discrete foci (83% co-staining of clone 6 and SBP1) (**Figure 4B**), resembling the typical labelling of proteins located in *P. falciparum* Maurer’s clefts. Both clone 6 and SBP1 were found to have similar localisation patterns in chronic-stage infected erythrocytes, where about 76% of the discrete foci detected were positive for both proteins. Some localisation of clone 6 was also observed close to the periphery of the iRBC in 91% of the examined discreet dots (**Figure 4B**), however our flow cytometry data on unfixed iRBC (**Figures 5B, D** **)** suggest that 9G8 is not externally located on the iRBC membrane at this stage of the infection. We have previously shown that S7 PIRs, at the chronic phase of infection, are found to be localised as granular structures across the iRBC cytosol and near the iRBC membrane (**Figures 2B**, **3A**). However, the use of anti-*Pb*SBP1 to identify *P. chabaudi* Maurer’s clefts indicates that their localisation does not change in iRBC at the acute and chronic infection (**Figure 4B**). These data suggest that S7 PIRs do not co-localise with the Maurer’s clefts in chronic-phase iRBC and are possibly released as infection progresses to the chronic-phase.

With progression to the chronic-phase of infection and during ring-to-trophozoite developmental transition, traces of L1 PIRs were seen in the host cell cytoplasm to co-localise with clone 6 following 9G8 labelling (**Figure 3A** **; bottom panel).** This suggests that Maurer’s clefts might also be involved in the trafficking of L1 PIRs but to a lesser extent compared to the S7 PIRs.

### 3.5 S7 and L1 PIRs Are Not Detected on the Surface of *P. chabaudi*-Infected Erythrocytes

It has been suggested that PIR proteins, similar to proteins of other *Plasmodium* multigene families (Niang et al., 2009; Niang et al., 2014; Saito et al., 2017; Kyes et al., 1999; Blythe et al., 2008), are present on or close to the surface of iRBC (Del Portillo et al., 2001; Cunningham et al., 2005; Pasini et al., 2013; Yam et al., 2016; Harrison et al., 2020). The distribution of flexible, sequence-variable loops across their structures is also characteristic of a protein family undergoing diversification to avoid immunoglobulin-mediated clearance (Harrison et al., 2020). As shown above (**Figures 2B**, **3A**), immunofluorescence studies of acetone:methanol fixed *P. chabaudi* RBC using the anti-S7 and anti-L1 peptide antisera showed that S7 appeared to be associated with vesicle-like structures in the iRBC cytoplasm and L1 PIRs with the PV, in both acute- and chronic-stage parasites. However, in *P. chabaudi* iRBC obtained from chronic infection, S7 PIRs were observed to localise in close proximity to the iRBC membrane suggesting they might be localised on the surface. Fixation can adversely destroy the integrity of the cell membrane and thus it is not possible to conclude that PIR proteins are expressed on the outer surface of the iRBC. Therefore, live imaging and flow cytometry analysis of *P. chabaudi* iRBC following labelling with the same peptide antisera were performed to investigate whether S7 and L1 PIRs are externally localised on the iRBC membrane (**Figure 5**).

Live-cell immunofluorescent imaging of approximately 500-600 iRBC, across two individual biological replicates, obtained from acute and chronic *P. chabaudi* infections, showed complete absence of S7 and L1 PIRs on the iRBC surface, following staining with the anti-S7 or anti-L1 peptide antisera (**Figure 5A**; top panels), confirming that the S7s and L1s recognised by these antisera are not associated with the outer iRBC plasma membrane at the trophozoite-stage. They seem rather to remain within the iRBC, as shown in fixed sample preparations (81.18 ± 0.10% SEM S7+ iRBC and 98.03 ± 0.21% SEM L1+ iRBC). Labelling with the 9G8 and anti-MSP1 monoclonal antibodies, known to specifically target proteins located within the iRBC (Holder et al., 1992; Hartz et al., 1993) (**Figure 4A**; bottom panels), revealed positive staining only of acetone:methanol fixed/permeabilised iRBC.

To increase detection sensitivity of PIRs, we also conducted flow cytometric analysis of *P. chabaudi* iRBC at the acute- and chronic-phases of infection. Live (unfixed) iRBC were stained with anti-S7 and anti-L1 peptide antisera and compared with the same antibody staining on acetone:methanol fixed/permeabilised iRBC (**Figures 5C, D** **;** **Supplementary Figure S5**). The gating strategy used for the flow cytometry analysis is described in **Supplementary Figure S1**. The contour plots clearly demonstrate that live iRBC (Hoechst+) are not stained positively for the anti-S7 or anti-L1 antisera (mean 99.3 ± 0.3% SEM S7 and 99.5 ± 0.2% SEM L1 acute-phase parasitised RBC; mean 99.4 ± 0.4% SEM S7 and 99.4 ± 0.4% SEM L1 chronic-phase parasitised iRBC), whereas almost the entire population of the fixed/permeabilised iRBC (Hoechst+) was stained positive for either the anti-S7 or anti-L1 peptide antisera (mean 99.7 ± 0.1% SEM S7 and 99.7 ± 0.2% SEM L1 acute-phase parasitised RBC; mean 99.0 ± 0.5% SEM S7 and 98.2 ± 1.5% SEM L1 chronic-phase parasitised RBC). As with IFAs, positive staining with the 9G8 and anti-MSP1 monoclonal antibodies was observed only on fixed/permeabilised iRBC (**Figure 5B**; bottom panels).

Therefore, no significant staining of live iRBC on IFA or by flow cytometry could be seen with either the anti-S7 or anti-L1 peptide antisera. By contrast, fixed/permeabilised iRBC demonstrated clear labelling of the internal S7 and L1 PIR proteins with anti-S7 and anti-L1 peptide antisera. These data confirm that the S7 and L1 PIRs as recognised by these antisera, are not exposed on the surface of live iRBC but rather localised within them.

### 3.6 Fractionation of *P. chabaudi* iRBC to Investigate PIRs Localisation

**Figure 6:**
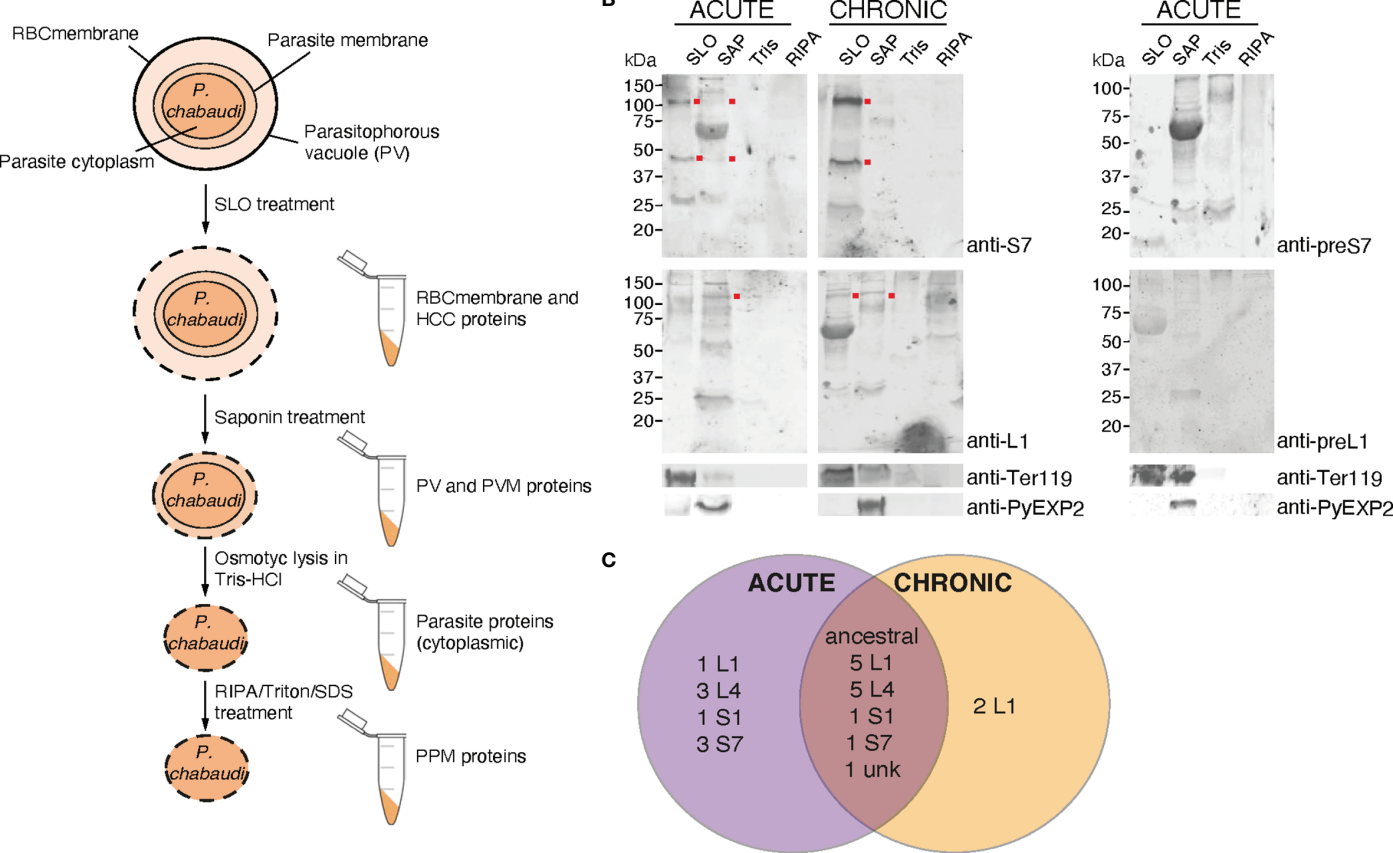
Subcellular fractionation of *P. c. chabaudi AS* iRBC and localisation pattern of the PIR proteins. **(A)** Schematic representation of the fractionation workflow used to map the subcellular localisation of *P. chabaudi* PIR proteins. Briefly, *P. chabaudi* iRBC at the trophozoite-stage were magnetically enriched and treated with SLO, prior to being centrifuged to obtain a supernatant and a pellet fraction. Proteins localised on the iRBC membrane and cytoplasm are expected to be found in the supernatant. The pellet was then treated with saponin to release all proteins localised on the PV and PVM. Following centrifugation, the resulting pellet was incubated with Tris-HCl hypotonic lysis buffer and frozen. Following osmotic shock of the cells, proteins localised within the parasite cytoplasm are expected to be released. The remaining parasite membrane-associated proteins were extracted by treating the resulting pellet with RIPA lysis buffer enriched with SDS and Triton. The lysed membrane following each treatment is represented by a dotted line. The composition of each pellet sample obtained is also indicated. RBCmembrane: Red Blood Cell membrane, PV; Parasitophorous Vacuole, PPM; Parasite Plasma Membrane, PVM; Parasitophorous Vacuole Membrane, HCC; Host-Cell Cytoplasm. **(B)** Western blot analysis of *P. chabaudi* trophozoite fractionated protein samples, under reducing conditions. Fractionated samples were prepared from infected blood of C57Bl/6 RAG1^-/-^ mice during acute- and chronic-phases of infection. Red dots indicate the estimated average size of the monomers or dimers of each PIR clade (S7 33-37 kDa and L1 75-151 kDa). Probing with pre-immune sera served as negative control. Antibodies against the mouse erythroid-specific marker Ter119 and *Plasmodium* EXP2 were used as stage-specific controls as well as to assess the samples purity. The molecular weights on the right indicate the positions to which size markers had migrated. SLO, Streptolysin-O fraction; SAP, Saponin fraction; Tris, Tris-HCl lysis. **(C)** Venn diagram depicting the overlap between the PIR proteins identified by mass spectrometry in all fractionated samples prepared from acute- and chronic-stage *P. chabaudi* infected erythrocytes. All PIR proteins identified in each set of samples are listed in **Tables 1** and **2**.

To further investigate PIRs subcellular localisation, membrane fractionations were carried out using samples of iRBC at the trophozoite-stage to separate proteins translocated to the periphery and the host-cell cytoplasm from those localised in the PV/PVM or those remaining in the internal of the parasite. **Figure 6A** illustrates schematically the strategy and workflow of the fractionation procedures employed to separate proteins associated with each subcellular compartment. Briefly, to isolate proteins localised on the iRBC membrane and host-cell cytoplasm, *P. chabaudi* MT acute- and chronic-stage erythrocytic parasites at the trophozoite-stage were magnetically enriched and treated with Streptolysin-O (SLO fraction). The main function of SLO is to cause haemolysis, and therefore to permeabilise the iRBC plasma membrane without affecting the PVM (Jackson et al., 2007; Ruecker et al., 2012). SLO-treated cells were then treated with saponin (SAP fraction), which disrupts the PVM without affecting the plasma membrane of the parasite. This allows extraction of proteins that are localised either on the PVM or within the PV. Subsequently, the membrane soluble fraction was extracted in a buffer containing Tris.HCl (Tris fraction), which causes osmotic lysis. Following this step, all proteins localised in the parasite cytoplasm are released. Lastly, hydrophobic proteins associated with the parasite plasma membrane and proteins that interact with other parasite plasma membrane proteins were extracted in a RIPA lysis buffer supplemented with additional SDS and Triton (RIPA fraction) (**Figure 6A**).

Samples collected as previously described were subjected to western blot analysis using the rabbit anti-S7 and anti-L1 peptide antisera (**Figure 6B**). The results showed that the antisera-specific S7 PIRs are present in the SLO-fraction, represented by bands that migrate at about 40 kDa and 100 kDa, meaning they are localised either on the host-cell membrane or cytoplasm. No differences in the localisation were observed between SBP acute and MT acute- or chronic-phase parasites. The weak signal observed in the SAP fraction of MT parasites might be related to carry over proteins from the SLO fraction, since a Ter119 faint band can be also observed in the SAP fraction. The size of S7 PIRs is predicted to be between 33-37 kDa, therefore the specific band observed at about 100 kDa suggests polymerisation. L1 PIRs have an expected size range between 75-151 kDa. Following western blot analysis of membrane fractionated samples of MT acute- or chronic- and SBP acute-phase parasites (**Supplementary Figure S6**), L1 antisera-specific PIRs were found to migrate at around 100 kDa and are predominantly present in the SAP fraction, where proteins associated with the PV and PVM are expected to be localised. In the MT chronic-phase parasites, a clear band was also observed in the SLO fraction. This agrees with the fluorescence microscopy data above (**Figures 2B**, **3A**), further suggesting secretion of L1 PIRs in the iRBC cytoplasm at this stage. The purity of the different samples was assessed by western blot probed with antibodies against the RBC membrane protein Ter119 and the PVM protein EXP2 (a gift of James Burns; Meibalan et al., 2015). The anti-EXP2 antibody revealed a ~30 kDa polypeptide, exclusively in the SAP fraction. Ter119 can be predominantly found in the SLO fraction, however some cross-contamination can be also observed in the SAP fraction. This is possibly associated with the high abundance of this protein on the RBC membrane, which is maintained in the sample after multiple washes, rather than RBC membrane debris contamination.

The anti-S7 and anti-L1 peptide antisera do not target all members of the PIR clades (**Supplementary Table S2**), therefore we performed mass spectrometry-based proteome analysis of the same fractionated samples (described above) as a more holistic approach to investigate the localisation of PIRs. Protein samples from three individual experiments were in-gel digested and analysed by LC-MS/MS using data independent acquisition. Acquired MS/MS spectra were then searched against databases of tryptic peptides predicted from all *P. chabaudi* as well as all host proteins. The results indicated that purity of the samples is likely to be of high quality since proteins whose location is known e.g. MSP1 (Holder et al., 1992), EXP2 (Meibalan et al., 2015), PCHAS_0624800 (Hartz et al., 1993), were identified in the corresponding fraction. Nevertheless, localisation of some of the recovered proteins indicated low-level contamination between serially prepared fractions. We compared the proteome of the acute-stage parasites with the proteome of the chronic-stage parasites to identify (1) PIR proteins that are highly expressed during the acute- and/or chronic-phases of blood-stage infection, and (2) their localisation on the iRBC (iRBC membrane/Host-Cell Cytoplasm (HCC), PV/PVM, Parasite Cytoplasm (PCyt) or Parasite Plasma Membrane (PPM)). PIR proteins identified in the fractionated samples are shown in **Table 1**, **2**. The full list of *P. chabaudi* and *Mus musculus* proteins identified is given in **Supplementary Table S3**.

**Table 1:** Localisation pattern of PIR proteins identified in proteome analyses of fractionated samples prepared from *P. chabaudi* infected erythrocytes at the acute phase of infection. .

Gene ID	Localization	Clade	AAPL/ChAPL Loci^1^	Chromosome
PCHAS_0101200	RBCmembrane/HCC, PV/PVM, PCyt, PPM	Ancestral		1
PCHAS_0200011	RBCmembrane/HCC, PV/PVM, PCyt, PPM	L4	AAPL	2
PCHAS_0200021	RBCmembrane/HCC, PV/PVM, PCyt, PPM	S1	AAPL	2
PCHAS_0200031	RBCmembrane/HCC, PV/PVM, PCyt, PPM	L4	AAPL	2
PCHAS_0300400	PV/PVM, PCyt	S7		3
PCHAS_0301400	RBCmembrane/HCC, PCyt, PPM	L1	ChAPL	3
PCHAS_0300900	PV/PVM, PPM	L1	ChAPL	3
PCHAS_0319600	RBCmembrane/HCC, PV/PVM, PPM	L4	AAPL	3
PCHAS_0319900	RBCmembrane/HCC, PV/PVM, PCyt, PPM	S7	AAPL	3
PCHAS_0320200	RBCmembrane/HCC, PV/PVM, PPM	S1	AAPL	3
PCHAS_0320000	PV/PVM, PCyt	S7	AAPL	3
PCHAS_0400500	PCyt	L4	AAPL	4
PCHAS_0524800	PPM	Unk		5
PCHAS_0600200	RBCmembrane/HCC, PCyt, PPM	S7		6
PCHAS_0600300	RBCmembrane/HCC, PV/PVM, PCyt, PPM	L4		6
PCHAS_0600500	RBCmembrane/HCC, PPM	L4		6
PCHAS_0600600	RBCmembrane/HCC, PV/PVM, PCyt, PPM	L4		6
PCHAS_0600700	PPM	L1		6
PCHAS_0600800	RBCmembrane/HCC, PV/PVM, PCyt, PPM	L1	ChAPL	6
PCHAS_0601100	RBCmembrane/HCC, PV/PVM, PCyt, PPM	L1	ChAPL	6
PCHAS_0626300	PV/PVM	L1	ChAPL	6
PCHAS_1200300	PPM	L4	AAPL	12

^1^AAPL/ChAPL classification was first referenced in Thibaut et al., 2017.

The localisation was defined based on which fractionated sample each PIR protein was recovered. RBC membrane, Red Blood Cell Membrane; HCC, Host-Cell Cytoplasm; PV, Parasitophorous Vacuole; PVM, Parasitophorous Vacuole Membrane; PCyt, Parasite Cytoplasm; PPM, Parasite Plasma Membrane.

**Table 2:** Localisation pattern of PIR proteins identified in proteome analyses of fractionated samples prepared from *P. chabaudi* infected erythrocytes at the chronic phase of infection.

Gene ID	Localization	Clade	AAPL/ChAPL Loci^1^	Chromosome
PCHAS_0101200	RBCmembrane/HCC, PV/PVM, PCyt, PPM	Ancestral		1
PCHAS_0200011	PPM	L4	AAPL	2
PCHAS_0200021	PPM	S1	AAPL	2
PCHAS_0200031	PCyt	L4	AAPL	2
PCHAS_0301400	PV/PVM, PCyt, PPM	L1	ChAPL	3
PCHAS_0524800	PPM	Unk		5
PCHAS_0600200	PPM	S7		6
PCHAS_0600300	RBCmembrane/HCC, PV/PVM, PCyt, PPM	L4		6
PCHAS_0600500	PPM	L4		6
PCHAS_0600600	RBCmembrane/HCC, PV/PVM, PCyt, PPM	L4		6
PCHAS_0600700	PCyt, PPM	L1		6
PCHAS_0600800	RBCmembrane/HCC, PV/PVM, PCyt, PPM	L1	ChAPL	6
PCHAS_0601000	PCyt, PPM	L1	ChAPL	6
PCHAS_0601100	RBCmembrane/HCC, PV/PVM, PCyt, PPM	L1	ChAPL	6
PCHAS_0626300	RBCmembrane/HCC, PV/PVM, PCyt, PPM	L1	ChAPL	6
PCHAS_0731300	RBCmembrane/HCC, PV/PVM, PCyt, PPM	L1		7

^1^AAPL/ChAPL classification was first referenced in Brugat et al., 2017.

The localisation was defined based on which fractionated sample each PIR protein was recovered. RBC membrane, Red Blood Cell Membrane; HCC, Host-Cell Cytoplasm; PV, Parasitophorous Vacuole; PVM, Parasitophorous Vacuole Membrane; PCyt, Parasite Cytoplasm; PPM, Parasite Plasma Membrane.

Proteomic analysis of four fractionated samples prepared from acute- or chronic-stage parasitised erythrocytes recovered on average 2,311 *P. chabaudi* and 3,634 *Mus musculus* proteins, across three individual biological replicates (**Supplementary Table S3**). Altogether, 23 individual PIRs were identified across all fractions of acute- and chronic-stage parasite preparations and all replicates conducted. Among them, 22 acute-stage associated PIRs and 16 chronic-stage associated PIRs were identified (**Table 1**, **2**, respectively). Among the PIRs recovered samples prepared from acute-stage parasites, 6 (27%) belong to the L1 clade, 8 (36%) to the L4 clade and 4 (18%) to the S7 clade. Additionally, 9 (41%) PIRs are located within the AAPL loci and 5 (23%) within the ChAPL loci. In total, 7 PIRs are located on chromosome 3 and 8 PIRs on chromosome 6. With regard to their subcellular localisation, 13 PIRs were picked up in at least 3 fractions suggesting that acute-associated PIRs are not limited to one subcellular compartment. Only 5 (23%) PIRs were exclusively found in one fractionated sample/subcellular compartment (1 in PV/PVM, 1 in PCyt and 3 in PPM). No association between clade or AAPL/ChAPL loci and subcellular compartment was found, however 14 PIRs were found to be localised in the iRBC membrane/HCC fraction, suggesting that they are exported proteins and further confirming our previous IFA findings (**Figure 2B**). Among the PIRs detected in samples prepared from parasites at the chronic phase of infection, only 1 (6%) of them belong to the S7 clade, 5 (31%) to the L4 clade and 7 (44%) to the L1 clade. Among the recovered PIRs, 3 (19%) are associated with the AAPL loci and 5 (31%) with the ChAPL loci. The majority of PIRs detected (9 PIRs; 56%) are located on chromosome 6, previously shown to be largely composed of ChAPL-associated clusters of *pir* genes (Brugat et al., 2017). Additionally, 7 PIRs (44%) were identified in all four subcellular compartments, 1 (6%) PIR in three subcellular compartments, and 8 PIRs (50%) were recovered exclusively within the parasite (either in PCyt or PPM). All identified PIRs, except PCHAS_0200031, were always being detected in the PPM fraction suggesting parasite membrane localisation within the iRBC. Comparing the PIRs identified at each phase of infection, a larger variation (e.g. AAPL/ChAPL loci association, chromosomal location, localisation pattern) and number of PIRs was recovered in acute-phase compared to chronic-phase parasite preparations (**Figure 6C**) possibly reflecting the complexity at this phase of infection.

Some of the PIRs detected in protein preparations of acute-phase parasites showed a wide distribution across all subcellular compartments, but as infection progresses to the chronic-phase, they appear to concentrate exclusively in one subcellular compartment e.g. PCHAS_0200011, PCHAS_0200021 and PCHAS_0200031 were recovered in all subcellular compartments of acute-phase parasites, but were not found to be secreted at the chronic-phase of infection.

The ancestral PIR, PCHAS_0101200 (Little et al., 2021), was consistently recovered in all samples prepared from both acute- and chronic-phase parasites and across all replicates conducted. Its high abundance in all subcellular compartments of the iRBC suggests that it is likely to be essential during parasite blood development.

In conclusion, there does not seem to be an exclusive subcellular compartment where PIR proteins are localised during both the acute- and chronic-phases of infection. This observation further supports the diverse nature of this family of proteins and consequently, the diversity in their function during *Plasmodium* blood development.

## 4 Discussion

The *pir* multigene family, present in the genomes of most species of the malaria parasite, *Plasmodium*, with the exception of members of the *Laverania* subgenus, has so far no known functions. Transcription of the genes is upregulated in the late liver-stages of the parasite in mice, and remains high in the asexual blood cycle (Spence et al., 2013; Brugat et al., 2017; Little et al., 2021) further suggesting they are important at these stages of the life cycle. As a prerequisite to understanding the role of *pirs* in the erythrocytic-stages of *Plasmodium* infection, we have carried out a detailed investigation of the location of *P. chabaudi* PIR proteins within iRBC of this rodent-infecting parasite using a variety of approaches: IFA, Electron microscopy, subcellular fractionation, western blot and mass spectrometry. *Pir* genes of *P. chabaudi* have been the most widely characterised (Lawton et al., 2012; Otto et al., 2014), which offers the possibility of studying their expression and role in acute and chronic infections (Brugat et al., 2017), and in genetically diverse strains of the parasites (Lin et al., 2017). Thus *P. chabaudi* can serve as a model for exploring their potential roles in human malarias caused by *P. vivax*, *P.* ovale, *P. malariae* and *P. knowlesi*.

We generated antisera specific for a large fraction of PIRs within each of the S7 and L1 clades, the two clades of the *pir* gene family predominantly transcribed in *P. chabaudi* (Otto et al., 2014; Brugat et al., 2017). In agreement with previous transcriptional data (Spence et al., 2013) MT blood-stages parasites seem to express more PIR proteins than SBP parasites (**Figure 2C**). By immunofluorescence assay and western blot analysis with mass spectrometry, both S7 and L1 PIR proteins are detected in iRBC; S7s are located as discrete foci in the cytoplasm of iRBC at the acute-phase of infection. L1 PIRs are localised close to the PV/PVM, with some expression in the iRBC cytoplasm at the chronic-phase of infection. For both S7 and L1 localisation does not change over the 24-hour life cycle in the iRBC; however, as infection progresses to the chronic-phase S7 PIRs are detected close to the periphery of iRBC suggesting they may be exported to the surface of the iRBC. *P. chabaudi* schizonts are sequestered during the infection in mice (Gilks et al., 1990; Brugat et al., 2014), and can only be obtained after a period of *in vitro* culture, which may affect the detection of PIR at that stage and improving the culture of *P. chabaudi* would clarify PIR expression at this stage of parasite development. The differential PIR protein expression shown here is similar to that described for PIR/CIR by Yam et al. (2016), by immunofluorescent assay, using antisera specific for either A or B PIRs, classified according to an earlier phylogenetic analysis of the encoded PIR proteins (Lawton et al., 2012), or GFP-tagged PIRs in transfected *P. chabaudi* parasites, where at trophozoite-stage most of the fluorescent signal was observed in the red cell cytosol, while at schizont-stage the signal was predominantly associated with individual merozoites. Another study using transgenic fluorescently tagged lines described PIR localisation as either patchy or punctate cytoplasmic, depending on the PIR, whereas in the liver most PIRs localised to the parasitophorous vacuole (Fougere et al., 2016). In agreement with our data, an earlier proteomic study also demonstrated an association of some PIRs with lipid rafts and trafficking beyond the PVM (Di Girolamo et al., 2008). Together these data suggest that PIR proteins of the different *pir* clades may have different functions.

Previously, we have shown that *P. chabaudi* parasites transmitted to mice *via* the mosquito vector are transcriptionally modified in asexual blood-stage parasite (Spence et al., 2013), and S7 *pir* genes are transcriptionally upregulated during the acute-phase of infection, whereas the chronic-phase is mostly dominated by the L1 *pirs* (Brugat et al., 2017). These S7 and L1 *pirs* were further categorised into virulence-associated clusters, called AAPL and ChAPL, respectively, as they occupy adjacent and distinct chromosomal loci, with L1 *pir* genes being particularly associated with chromosome 6 (Brugat et al., 2017). Here using mass spectrometry, we identify multiple PIR proteins of both S7 and L1 clades. While there is not a distinct expression profile of S7 or L1 and AAPL or ChAPL proteins at the acute-phase of *P. chabaudi* infections, as infection progresses to the chronic-phase, L1 PIRs seem to be predominantly expressed, with some of them belonging to the ChAPL loci, in agreement with the previous transcription data.

The observation of discrete foci of S7 staining at the acute- and chronic-phases of infection and L1 staining at the chronic-phase of infection in the iRBC cytoplasm, raises the possibility that S7s and L1s might be temporally associated with Maurer’s clefts. Maurer’s clefts are thought to function as sorting centres, through which *Plasmodium* proteins are trafficked on their way to the iRBC surface (Kriek et al., 2003; Tilley et al., 2008). The most important of these are surface parasite proteins involved in binding of iRBC to the host blood vessels, such as PfEMP1 encoded by the *var* multigene family, repetitive interspersed family proteins (RIFINs), and sub-telomeric variant open reading frame proteins (STEVORs), all of which localise to the Maurer’s clefts on their way to the iRBC surface (Kaviratne et al., 2002; Kriek et al., 2003; Haeggstrom et al., 2004; McRobert et al., 2004; Blythe et al., 2008). A number of other parasite proteins involved in modifying the host cell also localise to the Maurer’s clefts such as PfMC-2TMs (Tsarukyanova et al., 2009), several FIKK serine/threonine kinases (Nunes et al., 2007), as well as some members of the *Plasmodium* helical interspersed subtelomeric (PHIST) family of parasite proteins, with roles in host cell remodelling (reviewed by Warncke et al., 2016) and rigidity of the infected cell (Davies et al., 2020). Although the PEXEL/VTS protein export motif, a characteristic of many of the secreted proteins of *Plasmodium*, is absent from PIR proteins, they have transmembrane helices (Del Portillo et al., 2001; Janssen et al., 2004). Other Maurer’s cleft-associated proteins such as SBP1 and MAHRP1, which are localised on *P. falciparum* and *P. berghei* Maurer’s clefts, lack the PEXEL motif and are classified as PEXEL-negative exported proteins (PNEPs) (Blisnick et al., 2000; Spycher et al., 2003). SBP1 and MAHRP1 are part of the Maurer’s clefts trafficking machinery previously shown to be essential for the transport of PfEMP1 and other surface antigens to the iRBC surface. In *P. berghei* blood-stage parasites the PIRs analysed were found in the cytoplasm while in liver-stage parasites they remained associated with the parasitophorous vacuole (Fougere et al., 2016).

It is possible that the differential co-localisation of SBP1 and 9G8 may be due to different proteins being assembled prior to export, as described for proteins within J-dots, structures within the iRBC containing proteins for further export (Külzer et al., 2010; Kulzer et al., 2012) (i.e. some dots are double positive for SBP1 and 9G8, where others are single positive for either SBP1 or 9G8). Maurer’s clefts have previously been described in *P. chabaudi* iRBC (Lanners et al., 1999) and here electron microscopy revealed Maurer’s cleft-like structures in the cytoplasm of iRBC. As both the SBP1 and S7/L1 are rabbit antibodies, co-localisation studies could not be performed. However, the antigen recognised by 9G98 monoclonal antibodies, co-localises with both the Maurer’s cleft protein SBP1 and with S7 PIRs, strongly suggesting that S7s are present in Maurer’s cleft at least during the acute-stage of infection in the mouse. In chronic-phase parasites S7s move closer to the inner periphery of the iRBC membrane and are no longer co-localised with the Maurer’s clefts (based on the SBP1 localisation). Direct visualisation of S7 proteins within Maurer’s cleft clefts is ongoing.

The association of S7 PIRs with Maurer’s clefts and their later location close to the iRBC membrane would suggest that PIRs, like the RIFINs, STEVORs and PfEMP1 of *P. falciparum*, are being relocated to the iRBC membrane (Kaviratne et al., 2002; Kriek et al., 2003; Haeggstrom et al., 2004; McRobert et al., 2004; Blythe et al., 2008). These antigenically variant *P. falciparum* proteins have been shown to be at the surface of the iRBC, and are thought to be involved in immune evasion as well as adhering to host cells such as endothelium and red blood cells (reviewed by Kyes et al., 2001). However, despite an association with Maurer’s clefts, PIRs cannot readily be ascribed to these roles. Unlike PfEMP1, where a single iRBC expresses a single variant which would be required for successfully evading the host antibody response by antigenic variation, our IFA and previous studies by Yam et al. (2016) suggest that iRBC can simultaneously express both S7 and L1 (or A and B) PIRs. This is in agreement with the observations of the Franke-Fayard laboratory (Fougere et al., 2016) where fluorescent signal was observed from both tagged PIRs in a double PIR transgenic line, and also single cell transcriptional data using *P. berghei* (Howick et al., 2019) showing expression of more than one *pir*/PIR in an individual iRBC. Furthermore, although S7 PIRs were observed close the iRBC membrane, we were unable to detect either S7 or L1 PIRs on live iRBC suggesting that they are not on the outer iRBC membrane. As our antisera do not detect all members of each clade, it is possible that those PIRs that were not picked up by our antisera are exposed on the iRBC surface. Alternatively, PIRs may be exposed transiently at only a particular time point in the 24-hour cycle, which we may have missed. It is also possible that peptide antisera do not recognise the S7 and L1 PIRs in their natural form in live iRBCs, since the linear peptides, used for immunisation to generate the peptide antisera, might be exposed in denatured but not in the native protein structures.

As mentioned above, *P. chabaudi* schizonts sequester in tissues and organs (Gilks et al., 1990; Brugat et al., 2014) and thus are difficult to obtain *ex vivo*, and *in vitro* culture of *P. chabaudi* is so far not very successful. *In situ* tagging of individual *pirs* and development of culture conditions to yield good viable schizonts may provide unequivocal evidence of location on the surface of the iRBC.

If PIRs are not located on the outer surface of iRBC, then where and why are they being transported by Maurer’s clefts? This may be explained by the fact that they are components of the *Plasmodium* translocon of exported proteins (PTEX) (De Koning-Ward et al., 2009) and thus, they may act as adaptors or chaperones for the transport of other proteins to the iRBC surface, as it has been described for HSP101 in *P. falciparum* (Beck et al., 2014; Elsworth et al., 2014). They may also be part of the parasite machinery involved in cytoskeleton remodelling as it has been described for the FIKKs of *P. falciparum*, which are also transported in Maurer’s clefts, as mentioned above (Davies et al., 2020). If these are possibilities, one is left with the question of why there are so many PIRs and why are they so diverse?

*The P. chabaudi* model for investigating this family of genes that exist in many *Plasmodium* species will allow us to address these questions and their roles in acute and chronic infection. The elucidation of proteins which interact with proteins of this multigene family, particularly those PIRs identified by mass spectrometry, may shed some light on their function.

## Data Availability Statement

The datasets presented in this study can be found in online repositories. The names of the repository/repositories and accession number(s) can be found in the article/**Supplementary Material**.

## Ethics Statement

The animal study was reviewed and approved by The Francis Crick Institute Ethical Committee.

## Author Contributions

MG, DC, and JL designed the project, analysed the data, and wrote the manuscript. MG performed research with experimental help from DC, CH, AV, SA, AA, LS, FU, and LT. MB performed the mass-spectrometric analysis. AS carried out the electron microscopy imaging and analysis. TH and MH carried out recombinant protein expression and purification. JL and MH provided reagents/materials/analysis tools. All authors read, critically revised, and approved the final manuscript.

## Funding

This work was supported by the Francis Crick Institute which receives its core funding from the UK Medical Research Council (FC001101), Cancer Research UK (FC001101) and the Wellcome Trust (FC001101); JL is a Wellcome Trust Senior Investigator (grant reference WT101777MA). MH is a Wellcome Investigator (220797/Z/20/Z) and this work was funded by the Medical Research Council (MR/T000368/1).

## Conflict of Interest

The authors declare that the research was conducted in the absence of any commercial or financial relationships that could be construed as a potential conflict of interest.

## Publisher’s Note

All claims expressed in this article are solely those of the authors and do not necessarily represent those of their affiliated organizations, or those of the publisher, the editors and the reviewers. Any product that may be evaluated in this article, or claim that may be made by its manufacturer, is not guaranteed or endorsed by the publisher.

## Supplementary Material

Supplementary Figure S1 Representative flow cytometry plots describing the gating strategy followed to investigate the surface localisation of S7 or L1 PIRS in P. c. chabaudi AS iRBC.

Supplementary Figure S2 Subcellular localisation of S7 and L1 PIRs in the late trophozoite stage of MT and SBP P. c. chabaudi AS parasites, during the acute- and chronic-phases of infection.

Supplementary Figure S3 Localisation pattern of S7 and L1 PIRs across the 24-hour asexualblood cycle of MT P. c. chabaudi AS parasites, during acute- and chronic-phases of infection.

Supplementary Figure S4 Localisation pattern of S7 and L1 PIRs in in vitro SBP acute-stage P. c. chabaudi AS schizonts.

Supplementary Figure S5 Contour plots of each individual replicate of the flow cytometric analysis performed on live and acetone:methanol fixed (control) P. c. chabaudi AS iRBC, following labelling with the anti-S7 or anti-L1 peptide antisera.

Supplementary Figure S6 Western blot analysis of fractionated protein samples of P. c. chabaudi SBP acute-phase parasites at the trophozoite-stage.

Supplementary Table 1 P. chabaudi PIR clade peptide motifs and BlastP results against clade members.

Supplementary Table 2 P. chabaudi PIR proteins identified as reciprocal best hits of each motif designed against the S7 and L1 clades.

Supplementary Table 3 Mass spectrometry-based analysis of PIR expression and subcellular localization during acute and chronic phases of infection.
