# Supplementary material for: Differential Trafficking and Expression of PIR Proteins in Acute and Chronic Plasmodium Infections
Source: Front Cell Infect Microbiol. 2022 Jun 16;12:877253. doi: 10.3389/fcimb.2022.877253 (PMC9245118; doi:10.3389/fcimb.2022.877253)
Supplement: Supplementary Figure S1 — Representative flow cytometry plots describing the gating strategy followed to investigate the surface localisation of S7 or L1 PIRS in P. c. chabaudi AS iRBC. [file DataSheet_1.pdf]

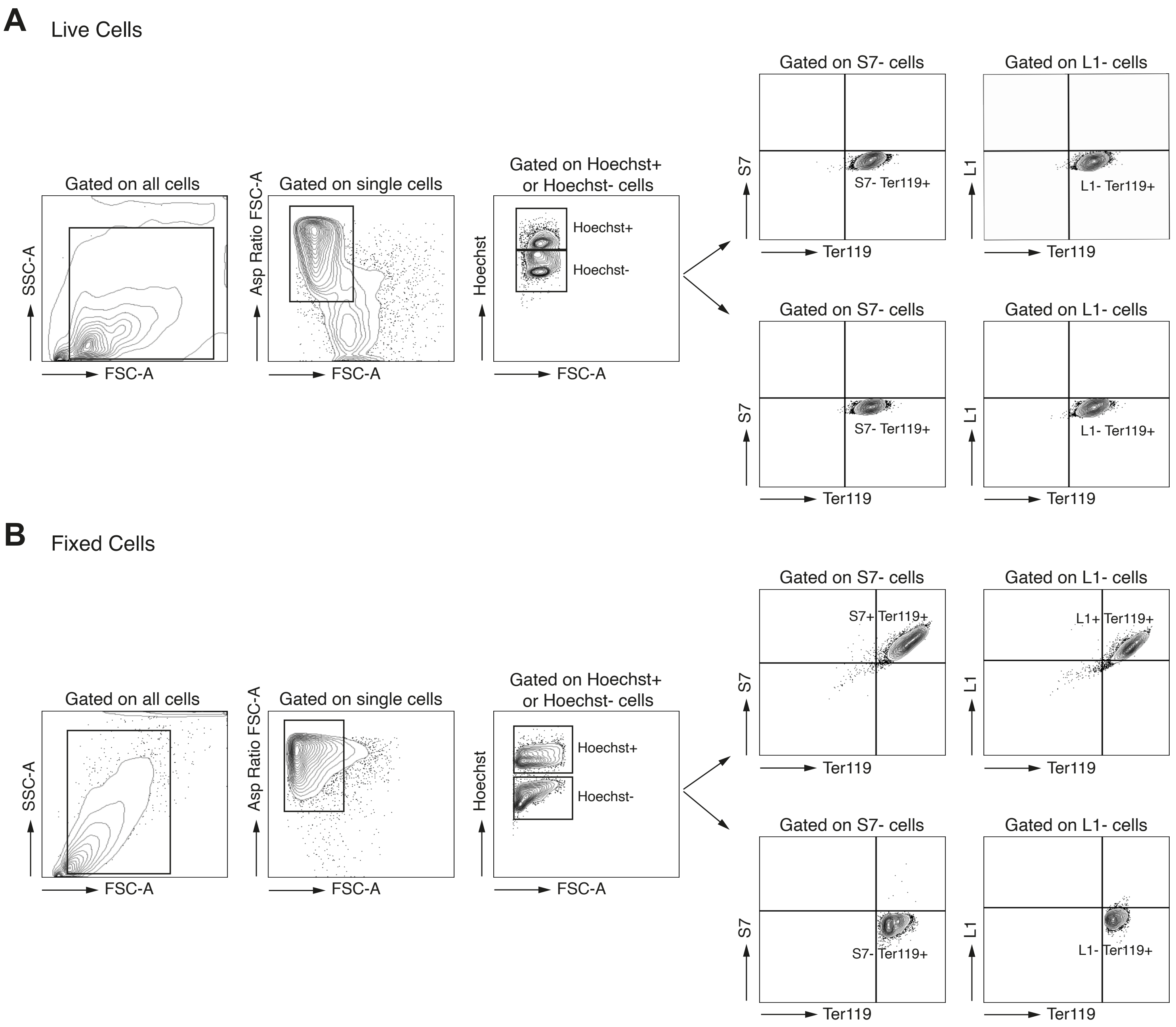
A
Live Cells
Gated on all cells
SSC-A
FSC-A
Gated on single cells
Asp Ratio FSC-A
FSC-A
Gated on Hoechst+
or Hoechst- cells
Hoechst
FSC-A
Hoechst+
Hoechst-
Gated on S7- cells
S7
Ter119
S7- Ter119+
Gated on L1- cells
L1
Ter119
L1- Ter119+
Gated on S7- cells
S7
Ter119
S7- Ter119+
Gated on L1- cells
L1
Ter119
L1- Ter119+
B
Fixed Cells
Gated on all cells
SSC-A
FSC-A
Gated on single cells
Asp Ratio FSC-A
FSC-A
Gated on Hoechst+
or Hoechst- cells
Hoechst
FSC-A
Hoechst+
Hoechst-
Gated on S7- cells
S7
Ter119
S7+ Ter119+
Gated on L1- cells
L1
Ter119
L1+ Ter119+
Gated on S7- cells
S7
Ter119
S7- Ter119+
Gated on L1- cells
L1
Ter119
L1- Ter119+

Supplementary Figure S1. Representative flow cytometry plots describing the gating strategy followed to investigate the surface localisation of S7 or L1 PIRS in *P. c. chabaudi AS* iRBC. Live (**A**) and fixed (**B**) iRBC from *P. chabaudi* infected C57BL/6 WT mice were labelled with the anti-S7 or anti-L1 peptide antisera and Ter119 monoclonal antibodies and analysed for mosquito transmitted parasites during the acute- and chronic-phases of infection in an Amnis® CellStream® Cytometer. Parasite DNA was stained with Hoechst 33342. Initially, the gate was set to exclude debris and include Hoechst+ iRBC based on the forward and side scatter (FSC-A/SSC-A) profile in the dot plot. The events were then viewed in an Aspect Ratio FSC-A/FSC-A profile to distinguish the singlet cells. The single cells were then gated into infected (Hoechst+) and non-infected (Hoechst-) RBC. Hoechst+ cells were then viewed in an S7 or L1/Ter119 dot plot. The S7 or L1 signal detected in Hoechst- cells was used as a control to set the baseline of the background coming from any non-specific binding of the peptide antisera. The fixative used for (A) is acetone:methanol which also permeabilises the cells; therefore fixed cells were used as a control to confirm antibody integrity.

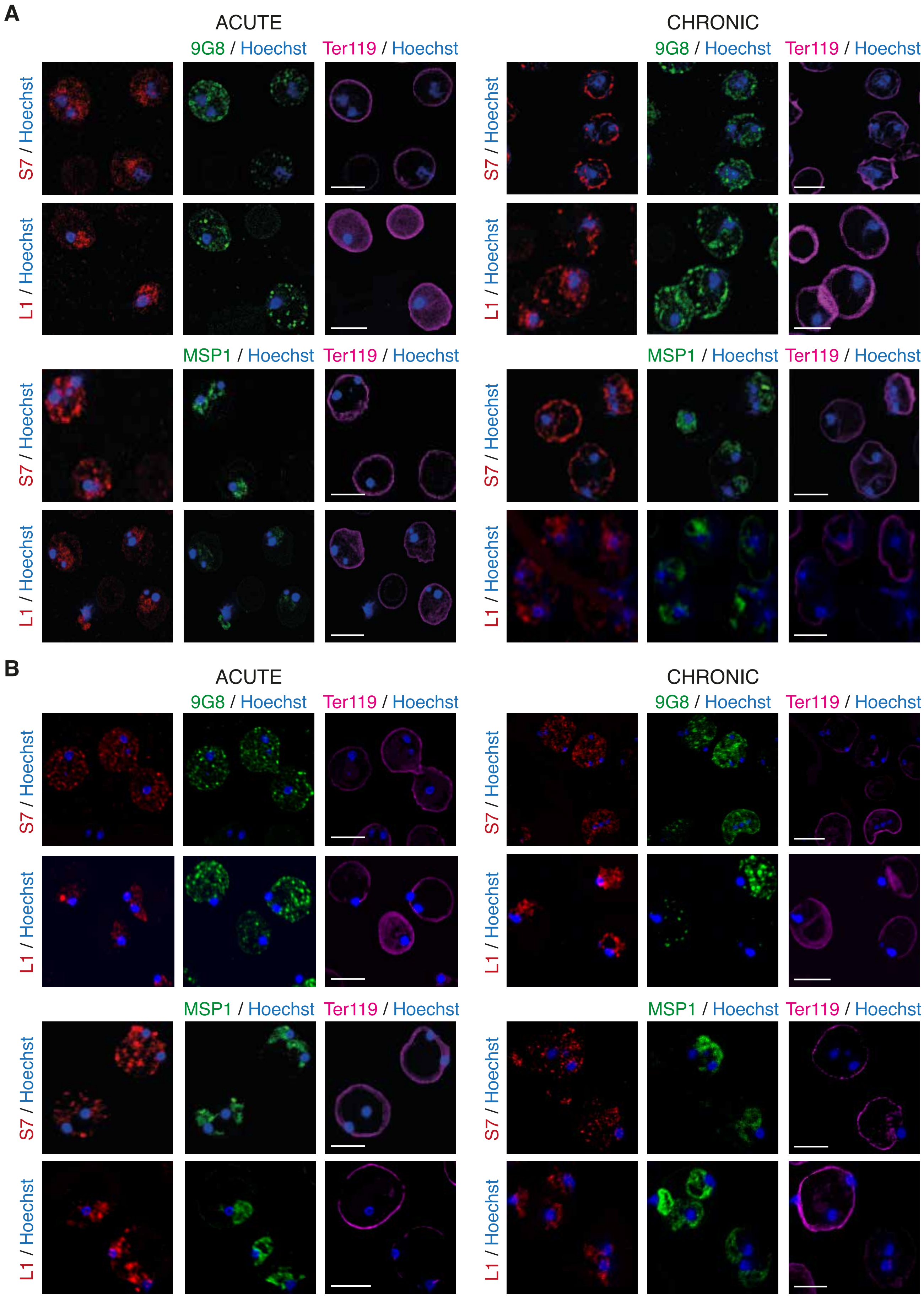
A
ACUTE
CHRONIC
9G8 / Hoechst
Ter119 / Hoechst
MSP1 / Hoechst
S7 / Hoechst
L1 / Hoechst
B
ACUTE
CHRONIC
9G8 / Hoechst
Ter119 / Hoechst
MSP1 / Hoechst
S7 / Hoechst
L1 / Hoechst

Supplementary Figure S2. Subcellular localisation of S7 and L1 PIRs in the late trophozoite-stage of MT and SBP *P. c. chabaudi AS* parasites, during the acute- and chronic-phases of infection. Single colour immunofluorescence images of MT (**A**) and SBP (**B**) WT *P. chabaudi* parasites at the late trophozoite-stage, isolated from C57Bl/6 RAG1$^{-/-}$ mouse blood. Parasites were primarily stained with the anti-S7 or anti-L1 peptide sera (red), and later with the anti-clone 6 (9G8) or anti-MSP1 monoclonal antibodies (green). The RBC surface membrane was stained with the rat anti-Ter119 monoclonal antibody (magenta), and parasite nuclei were stained with Hoechst (blue). Images were taken from confocal sections of acetone:methanol fixed parasites at X630 magnification. Scale bar length corresponds to 5 μm.

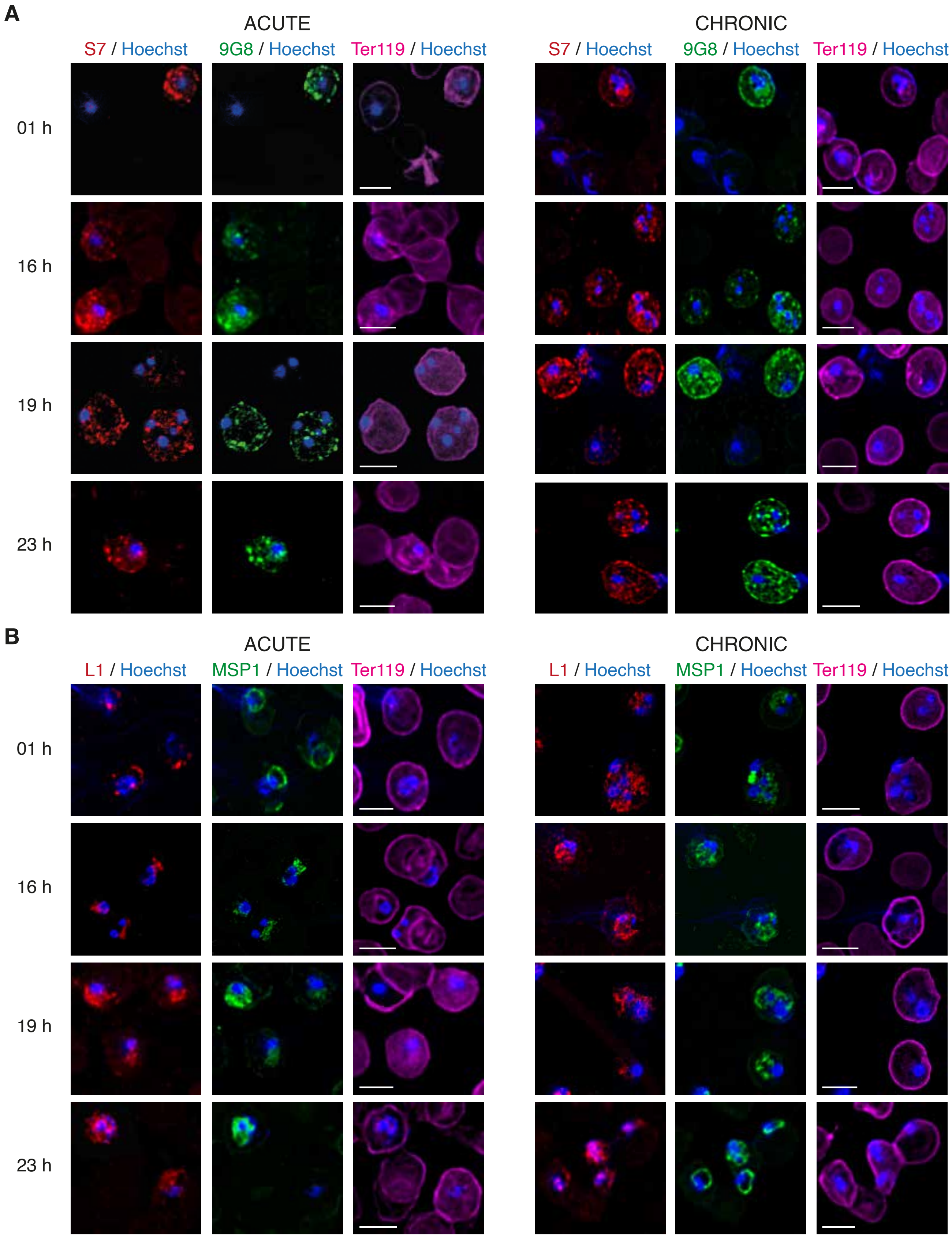
A
ACUTE
CHRONIC
S7 / Hoechst
9G8 / Hoechst
Ter119 / Hoechst
01 h
16 h
19 h
23 h
B
L1 / Hoechst
MSP1 / Hoechst
Ter119 / Hoechst

Supplementary Figure S3. Localisation pattern of S7 and L1 PIRs across the 24-hour asexual blood cycle of MT *P. c. chabaudi AS* parasites, during acute- and chronic-phases of infection. Single colour immunofluorescence images of MT WT *P. chabaudi* parasites. WT mice were directly exposed to infected mosquitoes and following infection establishment, parasites at the acute- (day-7 post infection) or chronic-phase (day-45 post infection) of infection were passaged into RAG1$^{-/-}$ mice, from which blood tail smears were prepared at the ring (01 h), early trophozoite (16 h), mature trophozoite (19 h), and late trophozoite/schizont (23 h) developmental stages. Parasites were primarily stained with anti-S7 (**A**) or anti-L1 peptide sera (**B**) (red). S7-incubated slides were then probed with the anti-clone 6 (9G8) (green) antibody, whereas L1-incubated slides were probed with the anti-MSP1 monoclonal antibody (green). The RBC surface membrane was stained with an anti-Ter119 antibody (magenta) and parasite nuclei were stained with Hoechst (blue). Images were taken from confocal sections of acetone:methanol fixed parasites at X630 magnification. Scale bar length correspond to 5 μm.

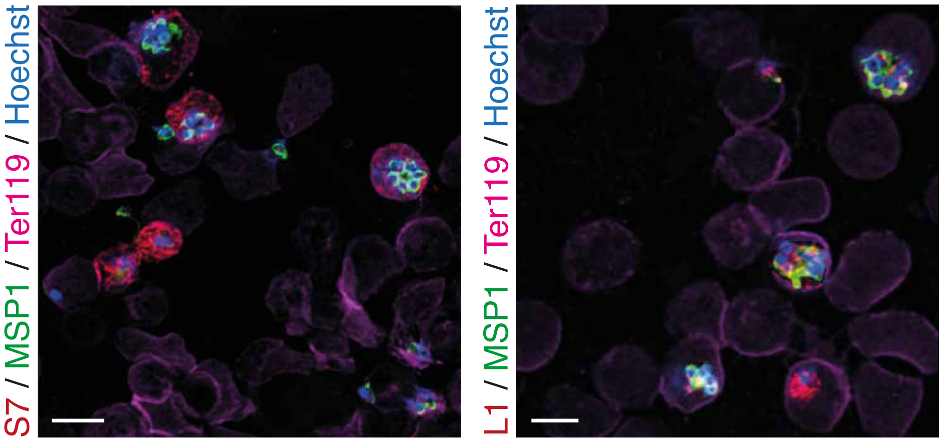

Supplementary Figure S4. Localisation pattern of S7 and L1 PIRs in *in vitro* SBP acute-stage *P. c. chabaudi AS* schizonts. Immunofluorescence assays of SBP acute-stage WT *P. chabaudi* parasites. Parasites were primarily stained with anti-S7 (left panel) or anti-L1 peptide sera (right panel) (red). S7- and L1-incubated slides were then probed against MSP1 (green), a prominent PVM protein. The RBC surface membrane was stained with an anti-Ter119 antibody (magenta) and parasite nuclei were stained with Hoechst (blue). Blood smears from *in vitro* schizont cultures were prepared following culture for five hours. Images were taken from confocal sections of acetone:methanol fixed parasites at X630 magnification. Scale bar length correspond to 5 µm. SBP; Serially Blood Passage.

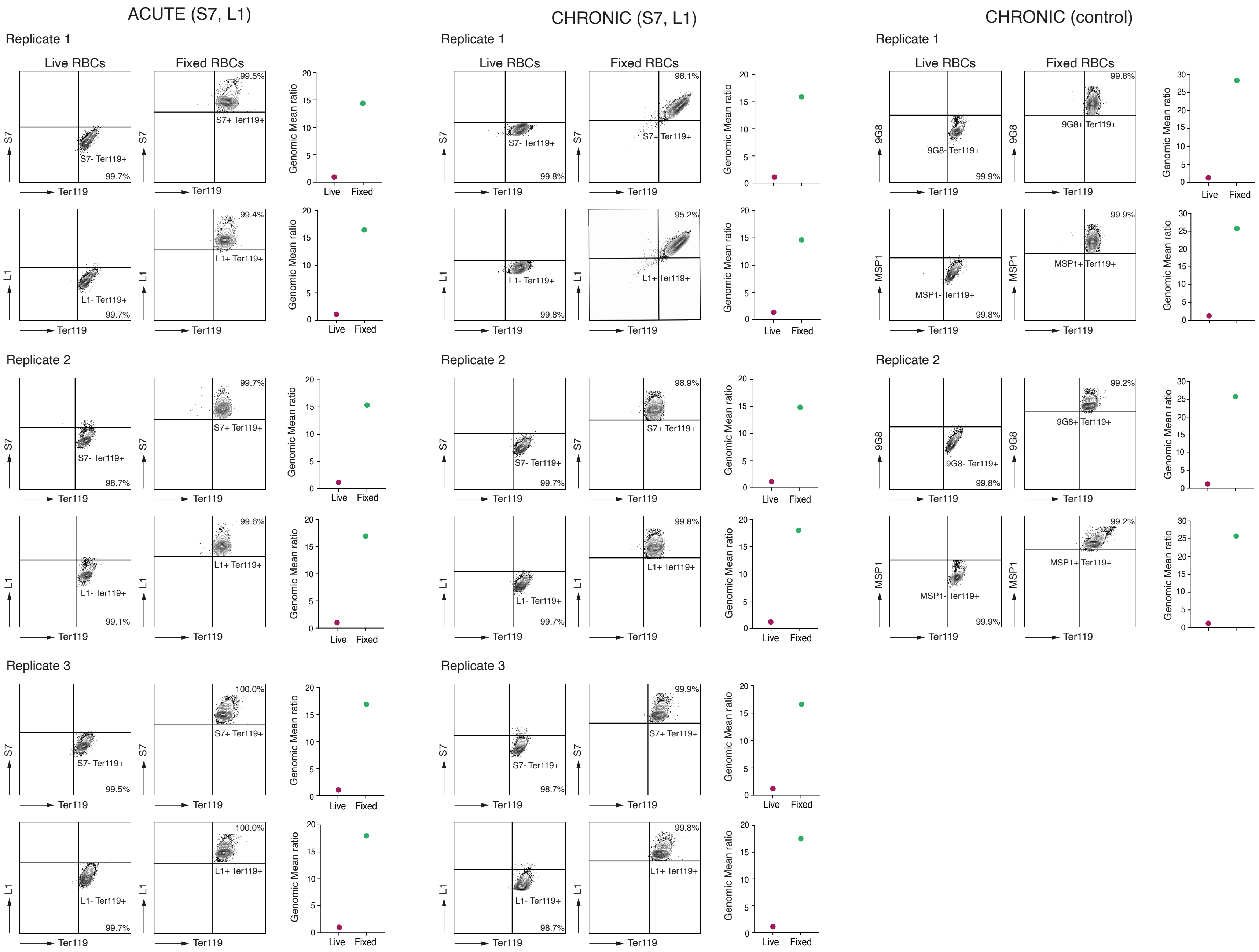
ACUTE (S7, L1)
CHRONIC (S7, L1)
CHRONIC (control)
Replicate 1
Live RBCs
Fixed RBCs
S7- Ter119+
99.7%
S7+ Ter119+
99.5%
L1- Ter119+
99.7%
L1+ Ter119+
99.4%
S7- Ter119+
99.8%
S7+ Ter119+
98.1%
L1- Ter119+
99.8%
L1+ Ter119+
95.2%
9G8- Ter119+
99.9%
9G8+ Ter119+
99.8%
MSP1- Ter119+
99.8%
MSP1+ Ter119+
99.9%
Replicate 2
S7- Ter119+
98.7%
S7+ Ter119+
99.7%
L1- Ter119+
99.1%
L1+ Ter119+
99.6%
S7- Ter119+
99.7%
S7+ Ter119+
98.9%
L1- Ter119+
99.7%
L1+ Ter119+
99.8%
9G8- Ter119+
99.8%
9G8+ Ter119+
99.2%
MSP1- Ter119+
99.9%
MSP1+ Ter119+
99.2%
Replicate 3
S7- Ter119+
99.5%
S7+ Ter119+
100.0%
L1- Ter119+
99.7%
L1+ Ter119+
100.0%
S7- Ter119+
98.7%
S7+ Ter119+
99.9%
L1- Ter119+
98.7%
L1+ Ter119+
99.8%
S7
L1
9G8
MSP1
Ter119
Genomic Mean ratio
Live
Fixed
0
5
10
15
20
25
30

Supplementary Figure S5. Contour plots of each individual replicate of the flow cytometric analysis performed on live and acetone:methanol fixed (control) *P. c. chabaudi AS* iRBC, following labelling with the anti-S7 or anti-L1 peptide antisera. Labelling with the 9G8 or anti-MSP1 monoclonal antibodies was used as a negative control for labelling integral parasite proteins in live cells, and as a positive control for staining integral parasite proteins in fixed/permeabilised cells. Erythrocytes were also stained with anti-Ter119, a monoclonal antibody that targets an abundant RBC membrane protein. One experiment with 3 mice per group for the anti-L1/anti-S7 labelling of acute and chronic parasitised iRBC, and 2 mice per group for the 9G8/anti-MSP1 labelling of chronic-phase parasitised iRBC was conducted. The side plots indicate the ratio of the geometric mean fluorescence intensity detected following binding of the anti-S7 or anti-L1 antibodies on Hoechst+ and Hoechst- live and fixed (control) RBC, for each individual replicate.

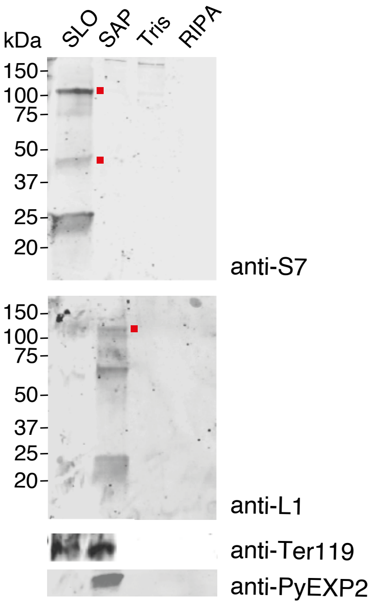

Supplementary Figure S6. Western blot analysis of fractionated protein samples of *P. chabaudi* SBP acute-phase parasites at the trophozoite-stage. Red dots indicate the estimated average size of the monomers or dimers of each PIR clade (S7: 33-45kDa; L1: 70-151kDa). Antibodies against the mouse erythroid-specific marker Ter119 and *Plasmodium* EXP2 were also used as stage-specific controls as well as to assess the sample purity. Western blot sample preparation was carried out under reducing conditions. The molecular weights on the right indicate the positions to which size markers had migrated. SLO; Streptolysin-O fraction; SAP; Saponin fraction; Tris; Tris-HCl lysis.

Supplementary Table S1. ***P. chabaudi*** **PIR clade peptide motifs and BlastP results against clade members.** Two peptide motifs were identified for each clade (motif 1 and 2). The number of BlastP results out of the total number of proteins belonging to each clade is indicated.

		**BlastP hits of peptide antisera against clade members**				
**Clade / Motif**	**Sequence**	**L1**	**S1**	**S3**	**S7**	**unk**
S7 Motif 1	CEKCSKDAKEFVNKYNELN	0	6 (16)	1 (3)	37 (70)	5 (5)
S7 Motif 2	CHSYDEMISSAVLFF	0	0	0	12 (70)	0
L1 Motif 1	CKTNYERINALGAYLY	49 (82)	0	0	1 (70)	0
L1 Motif 2	ACTLLREVDAYFNNE	29 (82)	0	0	0	0

Supplementary Table S2. ***P. chabaudi* PIR proteins identified as reciprocal best hits of each motif designed against the S7 and L1 clades.** The percent identity (%) between the peptide motifs and each PIR protein is indicated (BlastP E-value <0.01), as well as in which clade each PIR protein belongs to and whether it is associated with the AAPL or ChAPL loci. Protein hits with more than 70% similarity to the raised peptide motifs and E-value < 0.01 have been marked with an asterisk.

	Identity (%)	Clade	AAPL/ChAPL
**S7 Motif 1**			
PCHAS_0400300*	100	S7	AAPL
PCHAS_0300200*	74	S7	
PCHAS_0200015*	68	S7	AAPL
PCHAS_0600200	58	S7	
PCHAS_1371700	63	S7	
PCHAS_0320300	63	S7	
PCHAS_0627200	58	S7	
PCHAS_1147521	58	S7	
PCHAS_0300300	56	S7	
PCHAS_0200005	58	S7	
PCHAS_0115100	53	S7	
PCHAS_0213861	53	S7	
PCHAS_0400101	58	S7	
PCHAS_0500200	58	S7	
PCHAS_0732000	58	S7	
PCHAS_0938341	63	S7	
PCHAS_0320400	53	S7	
PCHAS_0938200	58	S7	
PCHAS_1147400	53	S1	
PCHAS_1400300	53	S1	
PCHAS_1401300	92	S1	
PCHAS_0800051	58	S1	
PCHAS_1371400	53	S7	AAPL
PCHAS_0300800	53	S7	
PCHAS_1147500	47	S7	
PCHAS_0320000	53	S7	AAPL
PCHAS_0400200	53	S7	AAPL
PCHAS_0900005	53	S7	
PCHAS_0400400	58	S7	AAPL
PCHAS_1371600	58	S7	AAPL
PCHAS_0100300	47	S7	
PCHAS_0319700	53	S7	AAPL
PCHAS_0900015	53	S7	
PCHAS_1247500	53	S7	
PCHAS_0700900	50	S7	
PCHAS_1200400	47	S7	AAPL
PCHAS_1300201	42	S7	
PCHAS_0319800	47	S7	AAPL
PCHAS_0731900	50	S1	
PCHAS_0114300	53	unk	
PCHAS_0207000	50	unk	

PCHAS_0900051	53	unk	
PCHAS_1406400	62	unk	
PCHAS_0405700	50	unk	
PCHAS_0700300	50	S1	
PCHAS_0800005	50	S7	
PCHAS_1400400	50	S1	
PCHAS_0115300	50	S7	
PCHAS_0525421	50	S3	
PCHAS_1371200	47	S7	AAPL
**S7 Motif 2**			
PCHAS_0400300*	100	S7	AAPL
PCHAS_0731800	67	S7	
PCHAS_0319700	73	S7	AAPL
PCHAS_0200025	80	S7	AAPL
PCHAS_1200700	67	S7	AAPL
PCHAS_0300800	53	S7	
PCHAS_0525411	67	S7	
PCHAS_1371200	67	S7	AAPL
PCHAS_1000400	67	S7	
PCHAS_0701300	53	S7	
PCHAS_0731600	59	S7	
PCHAS_0115300	62	S7	
**L1 Motif 1**			
PCHAS_0601600*	88	L1	
PCHAS_1146000*	88	L1	
PCHAS_0900100*	88	L1	
PCHAS_1302800*	75	L1	
PCHAS_1467900*	81	L1	
PCHAS_0800055*	81	L1	
PCHAS_0837200*	81	L1	
PCHAS_1001100*	81	L1	
PCHAS_0420700*	81	L1	
PCHAS_1467700*	75	L1	
PCHAS_0213821*	81	L1	
PCHAS_0700700*	75	L1	
PCHAS_0731500*	81	L1	
PCHAS_0113300*	73	L1	
PCHAS_0200055*	73	L1	
PCHAS_0900031*	75	L1	
PCHAS_1100300*	75	L1	
PCHAS_0601000	63	L1	ChAPL
PCHAS_1248300	69	L1	
PCHAS_0626800	67	L1	ChAPL
PCHAS_1401400	63	L1	
PCHAS_0400800	69	L1	
PCHAS_0500400	73	L1	
PCHAS_0837800	69	L1	

PCHAS_1468600	63	L1	
PCHAS_0100400	75	L1	
PCHAS_0600800	56	L1	ChAPL
PCHAS_0700400	80	L1	
PCHAS_1301700	63	L1	
PCHAS_0301700	60	L1	ChAPL
PCHAS_0800035	50	L1	
PCHAS_0200035	63	L1	
PCHAS_0701700	50	L1	
PCHAS_1000600	50	L1	
PCHAS_1370900	63	L1	
PCHAS_0419900	67	L1	
PCHAS_0601100	67	L1	ChAPL
PCHAS_0301400	60	L1	ChAPL
PCHAS_0419800	67	L1	
PCHAS_0700600	67	L1	
PCHAS_1147000	63	L1	
PCHAS_1302200	67	L1	
PCHAS_0114900	67	L1	
PCHAS_0300700	63	L1	
PCHAS_0302000	50	S7	ChAPL
PCHAS_1300601	67	L1	
PCHAS_0301900	60	L1	ChAPL
PCHAS_0626500	50	L1	ChAPL
PCHAS_0626600	67	L1	ChAPL
PCHAS_1370300	56	L1	
**L1 Motif 2**			
PCHAS_1100300*	100	L1	
PCHAS_0213821*	93	L1	
PCHAS_0100400*	86	L1	
PCHAS_0837800	64	L1	
PCHAS_0420700	73	L1	
PCHAS_0800055	67	L1	
PCHAS_0731500	60	L1	
PCHAS_1401400	60	L1	
PCHAS_0601600	64	L1	
PCHAS_0837200	64	L1	
PCHAS_1001100	64	L1	
PCHAS_1146000	69	L1	
PCHAS_1467900	64	L1	
PCHAS_0700700	64	L1	
PCHAS_1248300	64	L1	
PCHAS_1301700	64	L1	
PCHAS_1467700	60	L1	
PCHAS_0400800	64	L1	
PCHAS_0700400	57	L1	
PCHAS_1302800	57	L1	
PCHAS_0113300	60	L1	

PCHAS_0200035	57	L1	
PCHAS_0500400	57	L1	
PCHAS_0114800	57	L1	
PCHAS_0900031	57	L1	
PCHAS_1370900	57	L1	
PCHAS_1302200	50	L1	
PCHAS_0301600	47	L1	ChAPL
PCHAS_0419500	47	L1	
